# Organoids: From Bench to Bedside Applications

**DOI:** 10.1002/mco2.70768

**Published:** 2026-05-18

**Authors:** Kelin Li, Rui Cao, Maochen Li, Zichao Tian, Huahao Fan, Bixia Hong, Xiaojuan Liu

**Affiliations:** ^1^ College of Life Science and Technology Beijing University of Chemical Technology Beijing China; ^2^ School of Life Sciences Tianjin University Tianjin China; ^3^ School of Basic Medical Sciences State Key Laboratory of Respiratory Disease Guangzhou Medical University Guangzhou China; ^4^ Clinical Stem Cell Research Center Peking University Third Hospital Beijing China

**Keywords:** organoids, pathogenesis, stem cells

## Abstract

Organoids are three‐dimensiona(3D) models derived from stem cells that closely replicate the structure and cellular complexity of human tissues, providing physiologically relevant platforms for biomedical research. This technology addresses the limitations of two‐dimensional (2D) cultures, reduces species‐specific discrepancies, and is particularly valuable for investigating virus–host interactions and pathogenic mechanisms under near‐physiological conditions. This review systematically outlines key advancements in organoid‐based virology, including the propagation of hard‐to‐culture pathogens such as human rhinovirus C (HRV‐C) and norovirus (NoV), as well as novel insights into viral pathogenesis, including the severe acute respiratory syndrome coronavirus 2 (SARS‐CoV‐2) and Zika virus (ZIKV) infection, and the translational utility of organoids for antiviral drug screening and preclinical assessment. It further examines the use of organoids in modeling cancer and neurological diseases, compares the strengths and limitations of different cellular sources, and discusses their potential integration with emerging technologies such as CRISPR gene editing and 3D bioprinting. In addition, it maps a translational pathway from molecular mechanisms to clinical practice to facilitate the study of disease mechanisms and accelerate drug and vaccine development. Finally, holistic strategies are proposed to address existing challenges, such as the lack of immune components and inadequate vascularization. Together, these efforts aim to promote the broader adoption of organoid technology across the life sciences and translational medicine.

## Graphical abstract

Organoids, as a groundbreaking biomedical research platform, utilize adult stem cells (ASCs), embryonic stem cells (ESCs), and induced pluripotent stem cells (iPSCs) as sources. By integrating specific growth and differentiation signals within an extracellular matrix (e.g., Matrigel), organoids guide cells to self‐assemble into three‐dimensional microtissues with physiological structure and function. In virology, this technology simulates host–pathogen interactions, providing a dynamic model for elucidating infection mechanisms and antiviral drug screening. In oncology, patient‐derived organoids (PDOs) preserve tumor heterogeneity and are used to evaluate drug sensitivity, resistance mechanisms, and personalized treatment strategies. In neurology, brain organoids replicate nervous system development and disease states, offering a human‐specific model for studying neurodegenerative and neurodevelopmental disorders and potential therapeutic approaches. These applications collectively drive organoids from basic research toward clinical translation, supporting individualized diagnosis and treatment, discovery of novel biomarkers, and development of innovative drugs, ultimately establishing a complete translational pathway from bench to bedside.

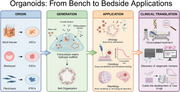

## 1 Introduction

The exploration of in vitro models that faithfully recapitulate human tissue physiology and pathological characteristics has long been a core pursuit in biomedical research. Traditional two‐dimensional (2D) cell culture lacks tissue heterogeneity and microenvironmental complexity. It cannot meet the needs of virus tropism research, which relies on tissue‐specific cells and microenvironments, or immune response research, which requires coordination between parenchymal cells and immune components. Interspecies differences limit the use of animal models, and their viral tropism spectra and immune response patterns do not match those of humans, leading to frequent failures in translating basic research into clinical practice. As a groundbreaking in vitro culture system, organoids have emerged as a bridge between basic research and clinical practice. Organoids are defined as three‐dimensional (3D) cellular aggregates derived from stem cells that can self‐organize and differentiate into structures that mimic the anatomical and functional features of corresponding in vivo organs [1, 2]. Organoids possess target‐organ‐specific multicellular composition, natural tissue‐like spatial organization, and tissue‐specific physiological and pathological simulation capabilities, offering significant advantages for virus tropism and immune response research. Organoids can accurately reproduce the invasion, replication, and spread of viruses in human target tissues, thereby overcoming the key limitations of 2D culture systems. By incorporating cocultured immune cells or retaining endogenous immune components, organoids can faithfully simulate human‐specific antiviral immune responses, overcome interspecies limitations inherent in animal models, and offer significant advantages over traditional models.

The development of organoid technology has witnessed several milestone discoveries since the 21st century. In 2009 [3], Hans Clevers’ team first successfully constructed intestinal organoids (IOs) from mouse intestinal stem cells, laying the foundation for establishing organoid culture systems. In 2013 [4], researchers further developed human IOs (HIOs), demonstrating the feasibility of using organoids in human‐related research. Subsequent years have seen continuous breakthroughs. For example, the successful construction of brain, liver, lung, and tumor organoids expanded the application scope of organoids to multiple tissues and diseases; the emergence of patient‐derived organoids (PDOs) in 2017 enabled personalized medicine research by retaining the genetic and pathological characteristics of individual patients; and the integration of organoids with microfluidic chips, single‐cell sequencing, and other technologies has further enhanced their simulation capability and research value. Despite these advances, research still faces challenges, including incomplete recapitulation of tissue complexity, high culture costs, and a lack of standardized experimental protocols, which limit the widespread clinical application of organoids.

There is an urgent need for more reliable models to clarify pathogenic mechanisms, screen therapeutic drugs, and develop personalized treatment strategies due to the increasing incidence of major diseases, such as infectious diseases, cancer, and neurological disorders. Existing reviews of organoids mostly focus on a single disease or technical principle and lack a comprehensive, systematic summary of their applications across multiple diseases, especially their translational value from basic research to clinical practice. Therefore, the aim of this review was to systematically synthesize the application progress of organoid technology in infectious diseases, cancer, and neurological diseases; analyze its role in resolving clinical bottlenecks such as unclear pathogenic mechanisms, inefficient drug development, and poor personalized treatment outcomes; and discuss current challenges and future development trends, thereby providing a comprehensive reference for researchers and clinicians in related fields.

## 2 History of Organoid Development

Organoids are complex cellular aggregates derived from embryonic stem cells (ESCs), induced pluripotent stem cells (iPSCs), and adult stem cells (ASCs) [5]. They exhibit the remarkable ability to replicate the cellular composition and functional characteristics of specific organs with high fidelity. This replication is achieved through 3D in vitro cultivation, in which cells undergo self‐assembly and self‐renewal under the influence of various inducing factors.

**FIGURE 1:**
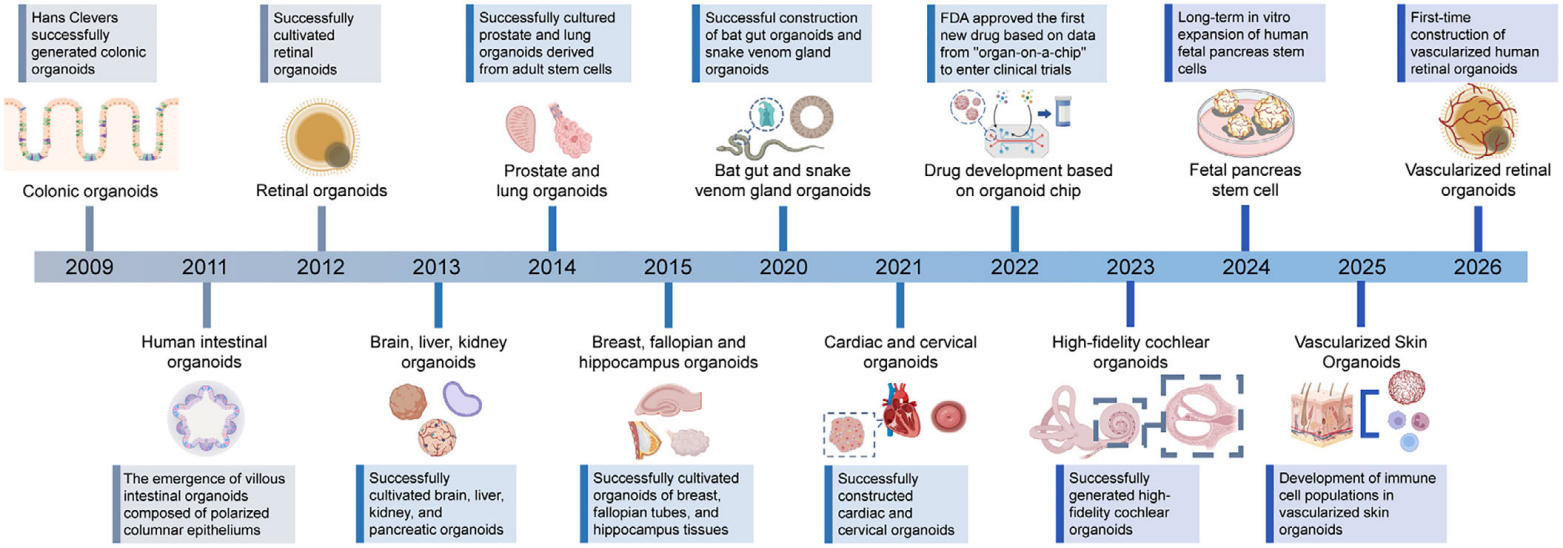
History of organoid development in the last decade. Since 2009, researchers have successfully cultivated multiple types of organoids, including colonic organoids (2009), human intestinal organoids (2011), retinal organoids (2012), brain, liver, kidney, and pancreatic organoids (2013), prostate and lung organoids (2014), breast, fallopian tube, and hippocampal organoids (2015), bat gut and snake venom gland organoids (2020), and cardiac and cervical organoids (2021). Following the United States Food and Drug Administration (US FDA)’s landmark approval of a trial drug using organ‐on‐a‐chip data in 2022, the field progressed toward higher physiological fidelity, generating functional cochlear organoids with hair cells (2023), establishing fetal pancreatic organoids that capture key lineages (2024), engineering vascularized skin organoids incorporating resident immunity (2025), and introducing transient vascular networks to alleviate chronic hypoxia in retinal organoids (2026). Created with BioRender.com.

Scientists began exploring methods to simulate the complex processes of organ development in vitro as early as the late 20th century [6, 7]. The pluripotency and self‐renewal capabilities of stem cells provided the foundation for generating organoids. Since Hans Clevers’ team achieved groundbreaking success in culturing IOs in 2009 [3], organoid technology has advanced rapidly (Figure 1), demonstrating immense potential for disease modeling, drug screening, and regenerative medicine. Building on this technology, iPSCs and ESCs have been further differentiated into various tissue‐specific organoids, such as heart, liver, lung, kidney, and pancreatic organoids, through the integration of advanced techniques [8]. Intestinal and retinal organoids were successfully cultured in vitro in 2011 [9, 10]. The IOs exhibited 3D villus‐like structures comprising polarized columnar epithelium, while the retinal organoids, derived from mouse ESCs, displayed characteristics of the retina and retinal pigment epithelium (RPE).

Researchers successfully generated organoids of the brain, liver, kidney, and pancreas from human pluripotent stem cells (hPSCs) in 2013 [4]. Prostate [11, 12] and lung organoids [13] were cultivated from ASCs by 2014, with fully differentiated CK5^+^ basal and CK8^+^ epithelial cells playing crucial roles in their formation. Mammary gland, fallopian tube [14], and hippocampal [15] organoids were successfully developed in 2015. The fallopian tube organoids contained both ciliated and secretory cells, marking a significant achievement in the field.

Our team established bat IOs in 2020, which were subsequently applied to study severe acute respiratory syndrome coronavirus 2 (SARS‐CoV‐2) infection. This development provided a critical tool for understanding virus–host tissue interactions and for developing potential therapeutic strategies [16]. Researchers expanded organoid technology to reptilian tissues in the same year by establishing long‐term cultures of venom gland organoids from several snake species [17]. Later, our team developed a biopotential lung organoid system capable of differentiating into airway and alveolar organoids under various conditions [18]. This work has significant implications for studying lung development, disease modeling, and regenerative medicine.

In 2021, Hans Clevers’ team achieved another milestone by pioneering the development of cervical organoids [19], which were subsequently used to study the female reproductive system. This groundbreaking innovation has provided a crucial tool for investigating cervical development, diseases such as cervical cancer, and infections such as human papillomavirus (HPV). Researchers also successfully created heart organoids [20], which are capable of replicating the early developmental stages of the human heart, in the same year. Further exploration of these heart organoids has unveiled the self‐organizing principles underlying human heart development, thereby offering novel tools and perspectives for the study of heart development, disease modeling, and drug development. The United States Food and Drug Administration (US FDA) approved the first new drug based on data from “organoid‐on‐a‐chip” to enter clinical trials in 2022 [21] (NCT04658472). This indicates that organoids may replace traditional models for drug evaluation.

Since 2022, a series of milestone advances have continuously expanded the application scope and significantly improved the physiological fidelity and functional precision of organoid technology. In 2023, Eri Hashino's team [22] established high‐fidelity cochlear organoids that faithfully recapitulate human auditory development, providing a robust experimental system for exploring the pathogenesis and treatment of hearing disorders. In 2024, expandable pancreatic organoids capable of differentiating into all pancreatic lineages were developed in Hans Clevers’ laboratory [23], offering a physiologically relevant platform for diabetes mechanistic study and preclinical drug discovery. The year 2025 marked the generation of the first fully vascularized and immunocompetent human skin organoid (SO) [24], which enables precise modeling of cutaneous tissue homeostasis and complex immune crosstalk. In 2026, Volker Busskamp's team [25] engineered vascularized retinal organoids to overcome chronic hypoxic stress, thereby alleviating retinal ganglion cell degeneration and facilitating the functional maturation of retinal tissues. Collectively, these iterative innovations have enhanced the physiological resemblance, cellular complexity and functional integrity of modern organoid models, consolidating their utility in developmental research, disease modeling, regenerative medicine, and translational pharmacology [26].

## 3 Construction of Organoids and Their Advantages and Limitations

**FIGURE 2:**
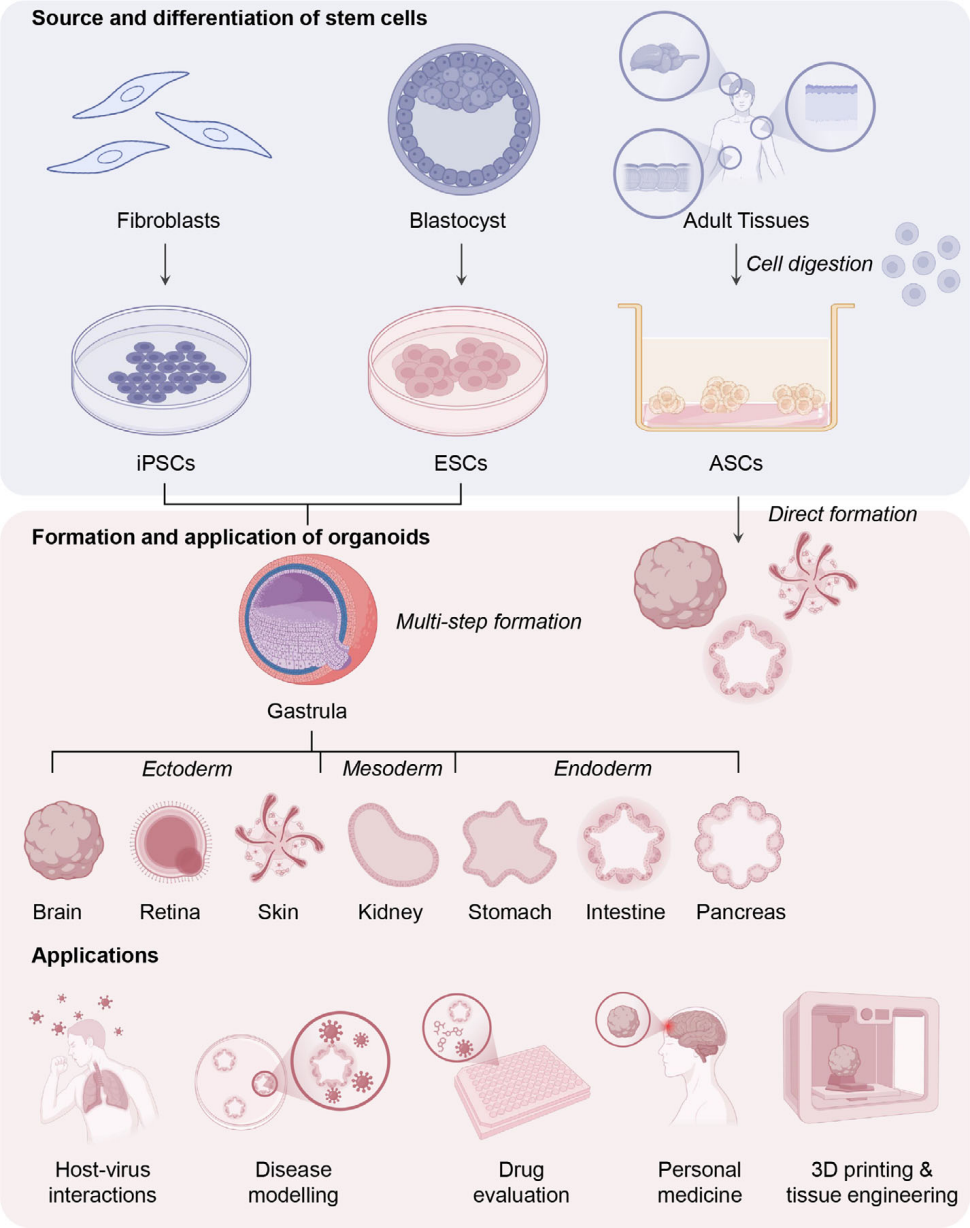
Origin and distribution of organoids. There are two types of organoids: one derived from induced pluripotent stem cells (iPSCs) and embryonic stem cells (ESCs), and the other derived from adult stem cells (ASCs). iPSCs, ESCs, and ASCs are derived from fibroblasts, blastocysts, and adult tissues, respectively. iPSCs and ESCs can differentiate into ectoderm, mesoderm, and endoderm through a multistep process, and then form organoids such as brain, retina, skin, kidney, stomach, intestine, and pancreas. ASCs can be induced to differentiate into corresponding organoids using specific culture media. Organoids from both sources have a wide range of applications in host–virus interactions, disease modeling, drug evaluation, personalized medicine, and 3D printing and tissue engineering. Created with BioRender.com.

Organoids can be derived from iPSCs, ESCs, and ASCs (Figure 2), each with distinct advantages, limitations, and applications (Table 1). iPSCs and ESCs are stem cells characterized by their remarkable potential to differentiate into diverse cell types. These cells can develop into nearly any cell type in the human body, including those originating from the three primary embryonic germ layers, that is, the endoderm, mesoderm, and ectoderm [27]. Inducing different organoids requires precise activation or inhibition of specific signaling combinations. For example, Shh signaling drives the formation of the ventral forebrain in neural organoids [28], and the combination of BMP4 and Wnt3a promotes cerebellar differentiation [29]; liver organoid construction often uses the “hepatoblast” strategy, relying on FGF/BMP signaling to coaggregate hepatic endoderm cells with mesenchymal and endothelial cells [30, 31, 32]; kidney organoids generally first induce mesoderm with Activin A [33, 34] and then induce intermediate mesoderm with FGF9 or BMP4 and FGF2 [33, 35]. Subsequently, retinoic acid, BMP2, and Activin A can be added to the mesoderm to induce ureteric buds [35], while retinoic acid and FGF9 are added during the intermediate mesoderm stage to induce metanephric mesenchyme [34]. Organoids derived from these stem cells can effectively model fetal organ development, making them particularly suitable for investigating the processes involved in organogenesis [36]. Their ability for unlimited proliferation and multilineage differentiation renders them invaluable for the generation of various tissues and organs, including the heart, liver, and small intestine [37, 38].

**TABLE 1:** Advantages and limitations of diverse stem cell‐derived organoids.

Sources	Advantages	Limitations	Applications
ESCs [39, 40]	Multidirectional differentiation potential; Infinite self‐renewal	Usually involves ethical issues; Risk of immune rejection at the time of transplantation; High cost	Developmental biology research, disease modeling, etc.
iPSCs [41, 42, 43]	Unlimited proliferation; Multidirectional differentiation potential; Genetically editable	The reprogramming process may affect cell function and stability; High cost; Complex culture conditions	Testing new drugs, personalizing treatments, studying mechanisms of reprogramming, etc.
ASCs [3, 44]	Wide range of sources; Long‐term amplification; Genomic stability	Limited differentiation capacity; Lack of nervous and vascular systems	Tissue transplantation and repair, testing new drugs and disease models, etc.

Abbreviations: ASCs, adult stem cells; ESCs, embryonic stem cells; iPSCs, induced pluripotent stem cells.

Another category of organoids is derived from ASCs. This class of organoids is relatively simple to construct and more readily available than those derived from iPSCs and ESCs. Although their culture systems vary depending on the tissue, they generally rely on the coordinated regulation of several core signaling pathways. The Wnt/β‐catenin pathway (commonly activated by agents such as R‐spondin‐1, Wnt3a, and CHIR99021) is key to maintaining stem cell self‐renewal [45, 46, 47, 48, 49]. At the same time, prodifferentiation signals must be inhibited, for example, by using Noggin, A83‐01, or SB431542 to block pathways such as BMP and TGFβ [46]. Additionally, FGF family members (e.g., FGF10) are often supplemented to further support cell growth and maintenance [41, 50]. Such organoids can recapitulate the developmental stages of their tissue of origin and closely resemble adult‐type organoids. In contrast, due to the embryonic origin of ESCs and iPSCs, the organoids derived from them are closer to fetal stages, and many physiological features have not yet matured [36]. Consequently, organoids derived from ASCs are more suitable for applications such as disease modeling, antiviral drug screening, testing new pharmaceuticals, and tissue repair [36, 51]. For instance, researchers can use intestinal samples derived from human tissues to construct disease models via CRISPR–Cas9‐mediated knockdown, facilitating the study of disease onset and progression [52]. Additionally, tissue‐derived animal IOs can accurately replicate the authentic animal intestinal epithelium, and their robust regenerative capacity reduces reliance on live animals [52, 53].

Experimental design necessitates the strategic selection of organoid models based on their features. ESCs are highly valued in tissue engineering due to their extensive plasticity and differentiation potential [54]. iPSCs circumvent ethical concerns and the risk of immune rejection, as they can be reprogrammed from adult cells [55]. ASCs are readily accessible and exhibit a low propensity for tumorigenesis [56]. Organoids derived from ASCs can be sourced from a wide array of parental tissues, facilitating the creation of a comprehensive library of biological samples [14, 50, 57]. Additionally, these cultures maintain genetic stability [58].

## 4 Multifield Applications of Organoids

With their unique ability to faithfully recapitulate the structural and functional characteristics of in vivo tissues, organoids have been widely applied across multiple biomedical fields. This chapter focuses on the multifield applications of organoids, systematically synthesizing their roles and research progress across core fields such as virology, oncology, and neuroscience. By integrating representative applications and clinical translation outcomes, it demonstrates the broad application value of organoids, providing a comprehensive reference for researchers in related fields.

### 4.1 Applications of Organoids in Virology Research

Recent breakthroughs in organoid technology have revolutionized virology research. Pathogens such as noroviruses (NoV) [59] and human rhinoviruses (HRV) [60], which are difficult to culture in conventional immortalized cell lines, can now be successfully propagated in organoid models. In addition, organoids can be used to simulate viral infection mechanisms, host–virus interactions, antiviral drug screening, and vaccine development. This technological advancement provides a physiologically relevant platform for studying viruses.

Organoids offer two distinct advantages over traditional research models. First, their surface molecule expression profiles more accurately recapitulate those of human tissues. The significant expression discrepancies observed in immortalized cell lines or animal models may lead to misinterpretation of viral receptor recognition mechanisms, particularly during laboratory adaptation processes [61]. Second, the unique polarized architecture of organoids serves as an irreplaceable model for studying viral strategies to breach epithelial barriers, a critical feature absent in immortalized cell lines [62, 63].

Among various organoid models, those derived from ASCs demonstrate distinctive advantages. These organoids fully retain the biological characteristics of epithelial cells (the primary targets of viral infection) and serve as an ideal platform for studying mechanisms of viral entry inhibition due to their short culture cycles and high differentiation potential [64, 65]. Currently, ASC‐based organoid models have shown significant value in virology research, particularly in advancing understanding of the infection mechanisms of major pathogens, including SARS‐CoV‐2, enteroviruses, Zika virus (ZIKV), and HRV. These studies have elucidated the molecular mechanisms of virus–host interactions and provided a crucial theoretical foundations for developing novel antiviral strategies.

#### 4.1.1 SARS‐CoV‐2

**FIGURE 3:**
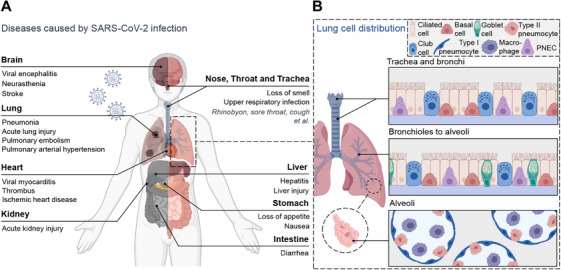
Symptoms of SARS‐CoV‐2 infection across organs of the human body and distribution of cells in different parts of the lung. (A) Infection of severe acute respiratory syndrome coronavirus 2 (SARS‐CoV‐2) can affect multiple organs, including the brain (viral encephalitis, necrotizing encephalitis), lungs (pneumonia, acute lung injury, pulmonary embolism, pulmonary arterial hypertension), heart (viral myocarditis, thrombosis, ischemic heart disease), and kidneys (acute kidney injury). In addition, infection may also cause symptoms such as loss of smell, liver damage, loss of appetite, and diarrhea. (B) Cell types and distribution in different parts of the lung. Created with BioRender.com.

The COVID‐19 pandemic precipitated a global public health crisis. Although prevention and control measures have since transitioned to routine management, several key clinical challenges continue to limit their effectiveness. These include real‐time monitoring of novel variants with immune‐escape capabilities, optimizing personalized treatment regimens across different severity strata, and achieving precise antigenic matching between vaccine strains and circulating variants. The clinical manifestations of SARS‐CoV‐2 infection are remarkably heterogeneous, ranging from predominantly upper respiratory symptoms such as sore throat, dry cough, and mild dyspnea in mild cases to rapid progression toward acute respiratory distress syndrome, septic shock, and multiorgan damage involving the heart, kidneys, and brain in severe cases [66, 67, 68]. This necessitates that diagnostic and therapeutic strategies be based on a precise understanding of its pathogenesis (Figure 3).

**FIGURE 4:**
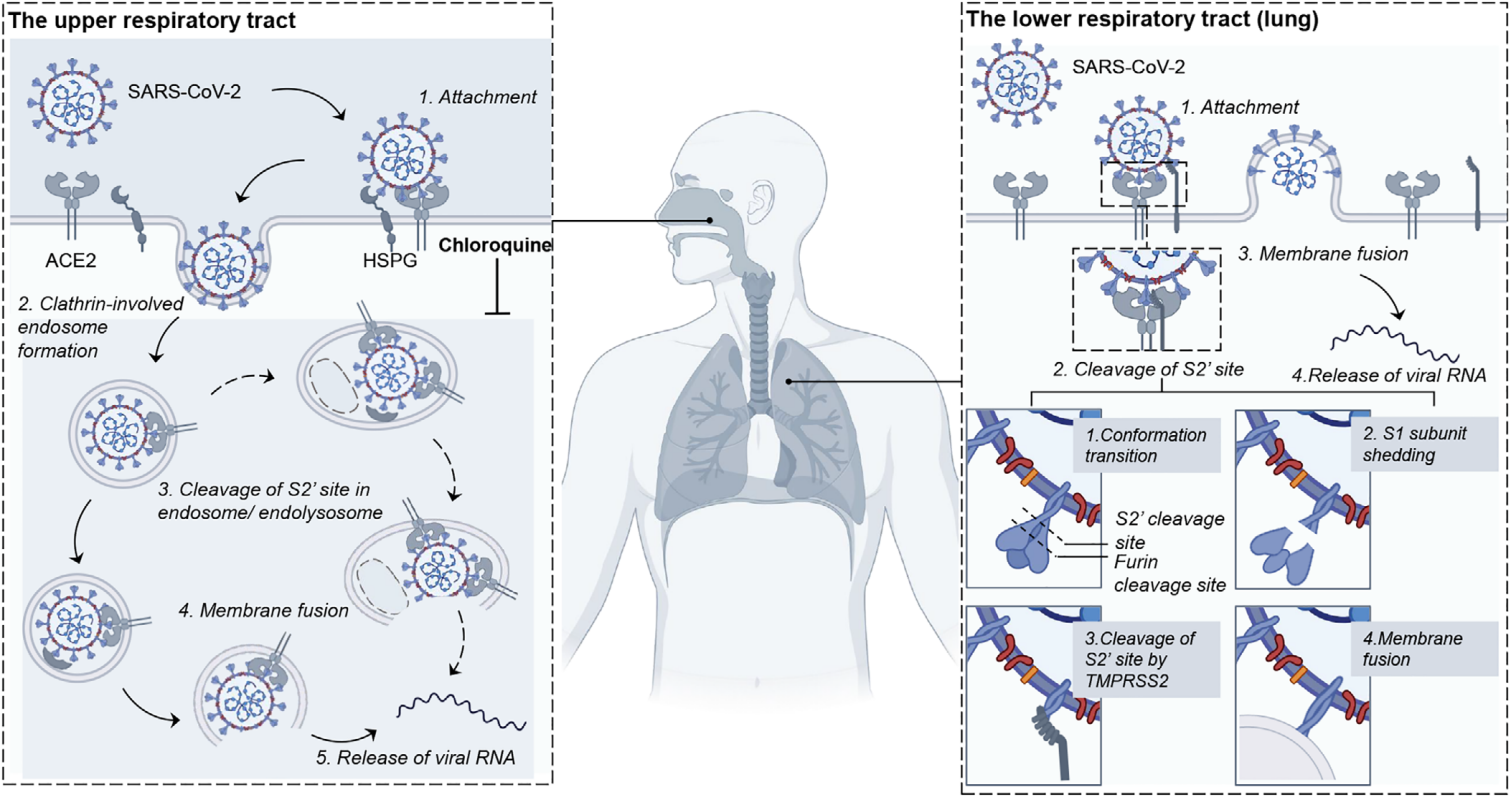
The entry mechanism of SARS‐CoV‐2 and the mode of action of chloroquine. After the SARS‐CoV‐2 spike (S) protein binds to ACE2 on the surface of the host cell membrane, there are two pathways: endocytosis and membrane fusion. In the endocytosis pathway, the S protein of the virus is cleaved by tissue protease CTSL, which is commonly found in upper respiratory cells. In the membrane fusion pathway, the S2 site of the viral spike protein is cleaved by transmembrane serine protease 2 (TMPRSS2), leading to the shedding of the S1 subunit and exposure of the S2 subunit, promoting the fusion of the viral membrane with the host cell membrane. This pathway is commonly found in lower respiratory cells. Chloroquine exerts its antiviral effect against SARS‐CoV‐2 by affecting the endocytosis pathway, but it cannot exert its antiviral effect in cells that rely on the membrane fusion pathway. Created with BioRender.com.

From the perspective of clinical therapeutic target selection, SARS‐CoV‐2 uses a dual‐entry mechanism, relying on both the angiotensin‐converting enzyme 2 (ACE2)/TMPRSS2‐mediated membrane fusion pathway and the cathepsin L (CTSL)‐dependent endocytic pathway [69] (Figure 4). This characteristic directly exposes the limitations of traditional single‐pathway, single‐cell‐line models in clinical translation. During the early pandemic, the unidirectional infectivity profiles of Vero cells (supporting only the CTSL‐dependent endocytic route) and Calu‐3 cells (supporting only the TMPRSS2‐mediated fusion route) led to the misinterpretation that chloroquine could achieve broad‐spectrum anti‐SARS‐CoV‐2 effects by blocking endocytosis. This prompted the premature launch of more than 10 global clinical trials of chloroquine therapy, which delayed the adoption of effective treatments and caused severe adverse reactions, such as cardiac arrhythmias in some patients [70, 71, 72, 73, 74, 75, 76, 77, 78]. This case highlights the inherent deficiencies of traditional models in simulating the complex human infection microenvironment and accurately predicting therapeutic efficacy.

A key clinical insight from organoid‐related research is the confirmation of the tissue‐specific distribution of SARS‐CoV‐2 entry pathways. In nasal mucosal organoids, viral entry primarily depends on the TMPRSS2‐mediated membrane fusion pathway (corresponding to mild upper respiratory tract infection). In contrast, in lower respiratory alveolar organoids, the CTSL‐dependent endocytic pathway predominates (corresponding to severe pulmonary infection). This distinction is particularly pronounced in lung tissues, especially within TMPRSS2‐low Type II alveolar epithelial cells, where the CTSL‐dependent pathway serves as the major entry mechanism [72, 73, 74]. The Omicron variant exhibits a shift in tropism, with reduced dependence on TMPRSS2 and increased reliance on CTSL [75]. Furthermore, clinical studies indicate that viral load positively correlates with CTSL expression levels but not with TMPRSS2 in lung tissue samples from deceased patients with COVID‐19 [79], providing further corroboration for this tissue‐specific pathway pattern.

The respiratory tract is the primary target organ for SARS‐CoV‐2. Its multilayered epithelial structure from the nasal mucosa to the alveoli, diverse cellular components [80] (e.g., ciliated cells, goblet cells, and Type II alveolar epithelial cells), and intact mucosal barrier necessitate that infection models faithfully recapitulate the human physiological microenvironment to accurately characterize the clinical disparities in infections at different sites. Lung organoids derived from ASCs can stably replicate the polarized structure and core physiological functions of the human lung epithelium, such as mucus secretion and ciliary beating, in vitro [81]. Furthermore, mature airway‐like and alveolar‐like organoid systems can simulate the cellular heterogeneity of lung tissue [18, 82, 83, 84, 85]. These advanced models have successfully identified the preferential infection of ciliated cells and the mechanisms of epithelial barrier disruption [86], providing crucial insights into the pathology of severe cases and accurately capturing the feature that the Omicron variant infects airway organoids 5–10 times more efficiently than the ancestral strain. The real‐time data generated offer vital evidence for variant surveillance, transmission risk assessment, and public health response.

More importantly, organoid models have revealed critical species‐specific differences in drug efficacy evaluation. For instance, the half‐maximal inhibitory concentration (IC_50_) of raltegravir in HIOs is 3.2 µM, approximately 70 times higher than the 46 nM measured in human colonic Caco‐2 cells [87]. This finding indicates that traditional cell models may substantially overestimate drug efficacy. Data generated from organoids exhibit greater physiological relevance, providing a more reliable basis for clinical dosing regimen design and efficacy prediction, thereby enhancing the overall efficiency of drug translation and development.

Despite their clinical value, the accuracy of organoids in simulating the in vivo infection microenvironment remains limited by the lack of immune components. Researchers have developed coculture systems combining lung organoids with macrophages differentiated from hPSCs to overcome this limitation [88]. This innovative approach offers a promising strategy for more accurately modeling physiological immune responses. The recently developed lymph‐node‐on‐a‐chip (LO chip) platform represents a breakthrough in clinical vaccine evaluation. This technology can simulate key immune response features of lymphoid tissues, including CD4^+^ T cell and B cell activation, plasmablast migration, and the uptake and expression of mRNA vaccines by myeloid cells [89]. By enabling precise immunogenicity analysis across different populations, this platform facilitates the development of personalized vaccination strategies and accelerates the optimization of novel vaccine candidates and their alignment with clinical applications.

#### 4.1.2 Enteroviruses

The enterovirus genus encompasses a variety of viruses, including coxsackieviruses, echoviruses, and polioviruses. Despite the widespread occurrence of enterovirus infections, understanding of their pathology and infection mechanisms remains limited.

In 2017, Coyne's team [90] successfully constructed HIOs using ESCs, which are pluripotent stem cells capable of differentiating into tissues derived from multiple germ layers. Phenotypic characterization confirmed that these organoids contain various intestinal cell types, including goblet, Paneth, and enteroendocrine cells, with a cellular composition achieving 72% concordance with that of human small intestinal tissue. Subsequent susceptibility studies further demonstrated that HIOs support infection by three clinically common enteroviruses: echovirus 11 (E11), coxsackievirus B (CVB), and enterovirus 71 (EV71) [91]. A key finding was the strict cell‐type specificity of viral infection within HIOs, with intestinal epithelial cells serving as the primary targets. Specifically, E11 preferentially infects absorptive enterocytes and enteroendocrine cells, thereby impairing intestinal absorption, whereas EV71 primarily replicates in goblet cells, leading to clinical symptoms such as diarrhea and abnormal mucus secretion [92]. These findings provide a crucial foundation for the precise classification and diagnosis of clinical enteroviral infections. By establishing organoids from endoscopic biopsy samples and detecting specific infected cell types, rapid pathogen identification can be achieved within 24 h.

The continuous optimization of organoid models demonstrates significant clinical application value at the preclinical and translational levels. Our team [93] developed an improved HIO by optimizing culture medium components to enhance physiological relevance. In this optimized model, intestinal epithelial cells constitute over 80% of the cellular population, while retaining four mature intestinal cell types, including goblet and Paneth cells. The infection characteristics of enteroviruses showed an increased concordance of 85% with intestinal tissues from pediatric patients, representing a substantial improvement in clinical relevance. Using this optimized system, we confirmed that infection with EV71 and coxsackievirus A16 rapidly activates signaling pathways involving IFN‐III, ISGs, and proinflammatory cytokines such as IL‐6 and TNF‐α. Pathway activity peaked at 72 h postinfection and subsequently declined gradually. This dynamic change elucidates the molecular mechanism underlying the self‐limiting nature of such intestinal infections. This discovery provides a scientific basis for clinical prognosis assessment. For example, measuring IFN‐III levels in intestinal tissue in patients could help predict whether an infection is likely to progress to a severe form, thereby helping avoid excessive antiviral treatment for mild cases. Furthermore, in translational drug development, Zhao's team [94] utilized HIOs to screen the protein kinase inhibitor GSK269962A. This compound inhibits viral replication in a dose‐dependent manner (IC_50_ = 0.8 µM) and shows no clear cytotoxicity in intestinal epithelial cells. Its pharmacodynamic profile provides guidance for designing clinical dosing regimens. Currently, this drug candidate has entered the preclinical safety evaluation stage for anti‐EV71 activity. Concurrently, research has identified Rock1 protein kinase as a novel host target for EV71. Small‐molecule inhibitors targeting this protein have completed in vitro activity validation. This discovery opens a new direction for developing specific anti‐EV71 drugs and accelerates the translational process from target discovery to preclinical validation.

Addressing the clinical challenge of preventing EV71 transmission via skin contact, a study used SOs (hiPSC‐SOs) to identify high mobility group box 1 (HMGB1) as a key receptor expressed on the surface of various skin cells, including keratinocytes and fibroblasts [95]. This finding clarifies the molecular mechanism of dermal transmission and provides a basis for optimizing contact isolation guidelines in hospitals and childcare settings. More importantly, targeted screening based on this receptor identified the inhibitor NSC167409. This candidate drug demonstrated three distinct effects in the SO model: it reduced viral load by over 85%, decreased IL‐1β levels by 60%, and improved abnormal progenitor cell proliferation. Furthermore, no significant skin irritation was observed in a mouse skin infection model. This candidate drug possesses antiviral and cytoprotective effects, representing a highly promising strategy for treating severe enteroviral infections. It has completed preclinical pharmacokinetic evaluation, laying the groundwork for subsequent clinical application.

#### 4.1.3 Hepatitis B Virus

Hepatitis B virus (HBV) poses a major global public health threat. According to WHO statistics, approximately 254 million people were living with chronic HBV infection worldwide in 2022, with 1.2 million new infections annually. HBV‐related deaths account for about 1.1 million per year, primarily due to liver cirrhosis and hepatocellular carcinoma [96]. However, due to its strict host specificity, the lack of research models that can accurately simulate the human infection state has long hindered progress in understanding its pathogenic mechanisms and in developing effective drugs. As a core pathogen causing liver cirrhosis and hepatocellular carcinoma, research on HBV has been slow due to the absence of suitable models [97, 98]. In recent years, liver organoid technology has successfully overcome many key limitations of traditional HBV research models. Currently, two highly promising research systems have been established: one is the iPSC‐derived liver organoids developed by the Nie team [99], and the other is the ASC‐derived liver organoids created by the de Crignis team [100]. Both systems can support the complete HBV lifecycle. Notably, ASC‐derived liver organoids can establish patient‐specific models and be reinfected with autologous viruses, making them valuable for screening unique host factors that influence individual HBV replication.

Liver organoids represent a revolutionary alternative to traditional preclinical research platforms, such as animal models and hepatoma cell lines, in HBV drug development. The case of Fialuridine exposed the fatal limitations of conventional research systems. Although this drug successfully passed all preclinical evaluations and demonstrated significant efficacy in reducing HBV DNA load, it caused severe hepatotoxicity in seven participants during Phase II clinical trials, leading to the termination of the study [101]. Subsequent investigations confirmed that its toxicity stemmed from interactions with nucleoside transporters specifically expressed in human mitochondria, a mechanism not detected in the nonhuman models and transformed cell lines used for prior testing. Notably, primary liver organoids exhibited significant and quantifiable toxic responses after treatment with Fialuridine [100], whereas the hepatoma‐derived HepG2 cells showed no adverse effects. This finding confirms that human liver organoids serve as a physiologically relevant preclinical platform for hepatotoxicity screening, effectively filling a critical gap in the traditional drug development pipeline.

#### 4.1.4 Human NoV

Human NoV (HuNoV) is a leading cause of both epidemic and sporadic acute gastroenteritis worldwide. The absence of approved antiviral therapies or vaccines against this pathogen constitutes a critical unmet clinical need. This therapeutic gap stems from the long‐standing lack of a stable and robust in vitro culture system. This has hindered fundamental studies on viral replication mechanisms, thereby creating a bottleneck in the translational pipeline for developing antivirals, vaccines, and elucidating infection pathogenesis [102, 103, 104].

A seminal advance was made by Mary et al. (2016) [59] with the establishment of HIOs that mimic intestinal cellular heterogeneity, enabling robust in vitro culture of HuNoV. Subsequent HIO‐based research has delineated strain‐specific entry pathways: GII.4 utilizes endosomal acidification via endocytosis, in which acidification of the endosomal compartment to pH ≈5.5–6.0 triggers conformational changes in the viral capsid, mediating the release of the viral genome into the cytoplasm [105], whereas GII.3 depends on bile acids and acid sphingomyelinase‐generated ceramides [106]. These discoveries have identified druggable host factors, thereby providing a molecular rationale for developing acidification inhibitors and acid sphingomyelinase modulators as antivirals. Clarification of these essential host dependencies also refines the selection of antigenic epitopes and the targeting of immune responses for vaccines, markedly increasing the precision of translational efforts against HuNoV.

#### 4.1.5 ZIKV

ZIKV is a flavivirus transmitted by mosquitoes and capable of vertical transmission. Due to its association with severe fetal neurological malformations and placental injury‐related developmental disorders, it poses a significant threat to global maternal and child health [107, 108, 109]. Despite its severe clinical outcomes, critical gaps remain in the development of precise prenatal risk assessment tools, the availability of specific antiviral therapies, and the implementation of targeted preventive strategies. In response to this escalating crisis, the WHO designated ZIKV as a Public Health Emergency of International Concern in 2016 [110], highlighting the urgent need to accelerate translational research related to ZIKV.

Researchers have constructed trophoblast organoids using human trophoblast stem cells, establishing a crucial model for ZIKV research. These organoids faithfully replicate core characteristics of natural trophoblast cells, including secretory functions, molecular expression profiles, and syncytialization, and have been shown to effectively model placental structural and functional damage caused by ZIKV infection [111]. Compared with traditional cell culture systems, these organoids more closely approximate the human physiological state, thereby establishing a reliable preclinical model for evaluating placental pathological damage. Following ZIKV infection, a decrease in syncytialization indicators, such as STB‐specific markers (CGB/hCGβ, syncytin, and multinucleation rate), serves as a quantifiable measure of placental dysfunction [111]. This improves the accuracy of predicting fetal infection risk and assessing placental dysfunction.

Cerebral organoids demonstrate significant translational value in clinical drug development and safety evaluation. Research platforms based on cerebral organoids have screened multiple promising candidate compounds, including JMX0207 [112], emricasan [113], betulinic acid [114], and TH6744 [115]. These compounds have shown both antiviral and neuroprotective effects in these models. Regarding their mechanisms of action, JMX0207 inhibits the viral NS3/NS2B protease complex [106], emricasan mitigates inflammation‐induced damage caused by infection, betulinic acid protects neural progenitor cells (NPCs) via the Akt pathway [107], and TH6744 blocks viral protein maturation by inhibiting Hsp70 activity [108]. The validation of the efficacy and safety of these compounds in cerebral organoids provides critical preclinical evidence for subsequent clinical trials, reducing the risks associated with further clinical testing and accelerating the translational research process.

Furthermore, studies using cerebral organoids have elucidated long‐term neuropathological mechanisms triggered by ZIKV infection, including dysregulated DNA methylation and FAM134B protein degradation [116]. These findings have directly informed the development of long‐term neurodevelopmental follow‐up protocols and the optimization of rehabilitation strategies for infected infants.

#### 4.1.6 Other Viruses

As an innovative and sophisticated model, organoids have been extensively utilized in the study of a diverse range of viruses (Table 2), including herpes simplex virus (HSV), HRV, rotavirus (RV), monkeypox virus (MPXV), and respiratory syncytial virus (RSV).

**TABLE 2:** Summary of organoid‐assisted virology studies.

Virus	Organoid type	Cell derived	Key findings
SARS‐CoV‐2	Kidney organoids [117]	iPSCs	SARS‐CoV‐2 infects and replicates in renal tubular cells, potentially explaining COVID‐19‐associated acute kidney injury.
	Heart and lung organoids [118]	hESCs	Androgen signaling is a key regulator of ACE2 levels.
Enterovirus	Forebrain organoids [119]	iPSCs	SH‐SY5Y cells are susceptible to EV‐D68; sialic acid facilitates organoid entry, but immune activation limits infection across developmental stages.
	Respiratory and intestinal organoids [120]	ASCs	NPEV‐C shows broad organoid tropism, whereas CVA‐13/CVA‐20/EV‐C99 selectively target airway ciliated cells.
ZIKV	Cerebral organoids [121]	iPSCs	SOAT1 maintains cholesterol balance essential for ZIKV morphogenesis and infection.
	Brain organoids [122]	hESCs	ZIKV depletes NPCs through TLR3, and TLR3 inhibition prevents this damage.
HSV‐1	Choroid plexus organoids [123]	iPSCs	Microglia curb HSV‐1 infection and maintain choroid plexus integrity through cGAS–STING‐driven interferon signaling.
	Early‐stage brain organoids [124]	iPSCs	HSV‐1 disrupts neural development, leading to stunted neurites and abnormal differentiation.
HSV‐1, HSV‐2, CMV	Trophoblast organoids [125]	iPSCs	DUX4 and DSGs are key antiviral defense mechanisms in trophoblast cells.
HPV	Cervical cancer organoids [126]	ASCs	HPV‐infected organoids recapitulate cervical precancerous progression with elevated Ki‐67 and p‐AKT expression.
	Ectocervix vorganoids [127]	ASCs	CT/HPV16 coinfection hijacks PI3K/Akt signaling and suppresses MAPK signaling to promote HPV persistence and cellular damage.
HBV	Liver organoids [100]	ASCs	HBV‐infected liver organoids show upregulated HCC‐associated genes (CCNA1, STMN2).
HRV	Respiratory organoids [60]	ASCs	Enable repeated culture difficult‐to‐culture HRV‐C and subsequent characterization of virus–host interactions.
RV	Porcine intestinal organoids [128]	Crypt cells	RV replication efficiency is significantly correlated with HBGA subtype.
HCV	Liver organoids [129]	iPSCs	HCV induces lipid accumulation via lipogenic pathways, while NAFLD‐driven lipid deposition and inflammation reciprocally enhance HCV replication.
	Liver organoids [130]	ASCs	HCV infection triggers CD8^+^ T cell‐mediated clearance of infected hepatic organoids.
MPXV	Skin organoids [131]	iPSCs	Tecovirimat blocks MPXV replication and host transcriptomic alterations in skin organoids.
	Colon organoids [132]	iPSCs	MPXV infection induces intestinal dysfunction in colonic organoids.
	Human neural organoids [133]	ESCs and iPSCs	MPXV lineage IIb causes neuronal degeneration and death by disrupting axonal transport, and the antiviral drug tecovirimat can significantly inhibit viral replication.
RSV	Bronchial organoids, nasal organoids [134]	ASCs	Nasal organoids exhibit higher RSV susceptibility than bronchial organoids, with enhanced postinfection immune responses and cytotoxicity.
	Human lung organoids [135]	iPSCs	RSV is vertically transmitted across the placental barrier and induces CC10 upregulation with FOXJ1 downregulation in human lung organoids.
HIV	Human cerebral organoids [136]	iPSCs	Human cerebral organoids derived from hiPSCs support HIV‐1 replication and gene expression.
	Intestinal organoids [137]	Intestinal epithelium‐derived cells and lamina propria‐derived cells	HIV‐1 can be internalized by intestinal epithelial cells and stored in LBPA‐enriched vesicles, after which mature viruses are released.

*Abbreviations*: ACE2, angiotensin‐converting enzyme 2; AKT, protein kinase B; ASCs, adult stem cells; CC10, club cell secretory protein; CCNA1, cyclin A1; CD8+, cluster of differentiation 8 positive; cGAS–STING, cyclic GMP–AMP synthase‐stimulator of interferon genes; CMV, cytomegalovirus; COVID‐19, coronavirus disease 2019; CT, Chlamydia trachomatis; CVA‐13/CVA‐20, coxsackievirus A13/A20; DSGs, desmogleins; DUX4, double homeobox 4; EV‐C99, enterovirus C99; EV‐D68, enterovirus D68; US FDA, Food and Drug Administration; FOXJ1, forkhead box J1; HBGA, histo‐blood group antigens; HBV, hepatitis B virus; HCC, hepatocellular carcinoma; HCV, hepatitis C virus; hESCs, human embryonic stem cells; HIV‐1, human immunodeficiency virus Type 1; HPV, human papillomavirus; HRV, human rhinovirus; HSV‐1/HSV‐2, herpes simplex virus Type 1/Type 2; Ki‐67, Kiel‐67 (a proliferation marker); LBPA, lysobisphosphatidic acid; MAPK, mitogen‐activated protein kinase; MPXV, monkeypox virus; NAFLD, nonalcoholic fatty liver disease; NPCs, neural progenitor cells; NPEV‐C, nonpolio enterovirus C; p‐AKT, phosphorylated AKT; PI3K, phosphatidylinositol 3‐kinase; RSV, respiratory syncytial virus; RV, rotavirus; SARS‐CoV‐2, severe acute respiratory syndrome coronavirus 2; SH‐SY5Y, Human Neuroblastoma Cell Line; SOAT1, sterol O‐acyltransferase 1; STMN2, stathmin‐2; TLR3, toll‐like receptor 3; ZIKV, Zika virus.

##### 4.1.6.1 HSV‐1

Studies on HSV‐1 infection have demonstrated that this virus can cause severe conditions, including keratitis, encephalitis, and neonatal herpes, with particularly serious effects in immunocompromised populations [138, 139]. Notably, iPSC‐derived brain organoids have revealed the capacity of HSV‐1 for efficient replication in brain tissue and its ability to induce specific neuroepithelial damage. Crucially, research has confirmed that IFN‐I effectively repairs such damage [97]. This discovery clarifies the viral mechanisms underlying cerebellar diseases and offers insights into host cellular immune defense networks.

##### 4.1.6.2 HRV

HRV is a primary causative agent of acute upper respiratory tract infections. However, since the identification of its subtype C (HRV‐C) in 2006, research on HRV‐C and HRV‐associated pathogenesis more broadly has been significantly hindered, primarily due to the difficulty of culturing HRV‐C in traditional cell lines [140, 141]. Nevertheless, recent breakthroughs have been achieved. Respiratory tract organoids support the stable passage of HRV‐C and have revealed that the immunosuppressant CYT387 can sustain its replication in airway organoids, while the virus completes replication spontaneously in nasal‐derived ASC organoids [60]. These findings address the long‐standing challenge of culturing HRV‐C and create new opportunities to investigate its transmission mechanisms and host–pathogen interactions.

##### 4.1.6.3 RV

As the primary global pathogen of viral gastroenteritis, RV has been the focus of significant advances in understanding its infection mechanisms and in the development of preventive strategies. Regarding pathogenesis, studies have confirmed that RV specifically recognizes host molecules on the surface of small intestinal epithelial cells via its VP4 protein. These molecules include glycans, histo‐blood group antigens (HBGA), and sialic acids (SA) [142, 143, 144]. Research utilizing organoids further revealed a significant correlation between RV replication efficiency and HBGA subtypes in porcine IOs. Organoids carrying HBGA type A exhibited a higher infection rate than those carrying type O [128]. Additionally, terminal SA modifications have a bidirectional regulatory effect on the infectivity of different RV strains [145]. These discoveries deepen the understanding of virus–host interaction mechanisms and provide a scientific basis for formulating personalized vaccination strategies, such as optimizing vaccine coverage based on HBGA subtypes within specific populations.

##### 4.1.6.4 MPXV

In recent years, organoids have emerged as a valuable platform for investigating the infection mechanisms of MPXV and its interactions with the host. Studies utilizing hPSC‐derived skin, colon, and neural organoids have progressively revealed the tissue‐specific infection characteristics and host response patterns of MPXV. Research indicates that human SOs are highly susceptible to MPXV infection. The virus primarily replicates within keratinocytes, undergoes typical intracellular assembly stages, and induces significant host transcriptional reprogramming. Early intervention with tecovirimat effectively inhibits viral replication and mitigates infection‐induced transcriptional alterations [131]. The antiviral efficacy of tecovirimat was further validated in neural organoid models [133].

MPXV infection in colon organoids leads to significant upregulation of stress response genes, such as HSPA6 and FOS, while downregulating genes associated with cytoskeletal organization and vesicular transport, including PSAP and CFL1. Clade I virus infection induces the most extensive transcriptional changes, suggesting potential disruption of epithelial integrity and protein folding homeostasis, which may correlate with gastrointestinal symptoms [132]. Furthermore, studies using neural organoids demonstrate that MPXV can infect NPCs and mature neurons. The virus spreads intercellularly, leading to the formation of viral factories, the appearance of beaded neurite structures, and neuronal degeneration, accompanied by upregulated expression of neurodegeneration‐associated transcripts. Treatment with tecovirimat significantly reduces viral load [133]. In summary, organoids provide a powerful tool for elucidating MPXV tropism, replication dynamics, host responses, and antiviral drug evaluation, thereby enhancing understanding of its multitissue pathogenesis and supporting the development of targeted therapeutic strategies.

##### 4.1.6.5 RSV

RSV is the leading causative agent of acute lower respiratory tract infections in children worldwide, resulting in an estimated 3.6 million hospitalizations annually [146]. Recent advancements in multicellular respiratory organoid models now enable reliable simulation of key features of RSV infection, including high expression of viral genomes and proteins, disruption of the epithelial layer, and increased collagen deposition. These models have been validated for evaluating the efficacy of antiviral drugs and monoclonal antibodies [147]. Furthermore, organoid‐based research provides novel insights into the cell‐type specificity of RSV infection, age‐related susceptibility, and immune regulatory mechanisms. Comparative studies using nasal organoids derived from infants and adults revealed that infant‐derived organoids exhibit greater viral susceptibility, stronger cytokine release, increased mucus production, and more severe epithelial damage after RSV infection. In contrast, the cytokine response in adult‐derived organoids is more controlled. This suggests that age‐specific epithelial immune characteristics may contribute to the more severe disease observed in infants [134]. RSV infection disrupts lung tissue structure development in human lung organoids in a dose‐ and time‐dependent manner. This is characterized by upregulated expression of the club cell marker CC10, downregulated expression of the ciliated cell marker FOXJ1, cytoskeletal rearrangement, and increased expression of the transient receptor potential vanilloid 1 channel. These findings indicate that fetal exposure to RSV may alter long‐term airway function [135]. Additionally, extracellular vesicles (EVs) isolated from the nasal secretions of RSV‐infected children and infected organoids were enriched with specific chemokines, such as CCL20 and CXCL8, and piRNAs. This reveals a mechanism by which RSV can modulate the local microenvironment and immune response via EV cargo [148]. Collectively, organoid models provide a powerful platform for the multidimensional analysis of RSV infection mechanisms, age‐related susceptibility, and potential therapeutic targets. This provides a critical reference for RSV pathogenesis research and the development of targeted intervention strategies.

The above studies have clarified the core role of organoid models in elucidating the characteristics of viral infection, and the translation of basic research into clinical practice depends on identifying and validating key targets. We summarize the key targets, mechanisms of action, and research progress across different stages of viral infection to further clarify the correspondence between targets identified in organoid research and clinical targeting strategies (Table 3). Organoid models can efficiently verify the efficacy and specificity of clinical candidate drugs and targets, providing crucial preliminary evidence for antiviral clinical translation. Beyond virology, organoids are also valuable in target discovery and therapeutic validation in oncology research.

**TABLE 3:** Summary of antiviral drug action pathways and translational evidence.

Pathway	Target	Mechanism of action	Clinical evidence	Organoid evidence	References
Host‐factor	Nrf2	Activates the Nrf2 pathway to counteract viral infection‐induced oxidative stress and mitochondrial impairment	Nrf2 pathway activation ameliorates virus‐induced oxidative stress and mitochondrial dysfunction.	Omaveloxolone protects liver organoids from dengue virus‐induced damage, indicating host‐mediated broad‐spectrum potential.	[149]
Entry/Host‐factor	TMPRSS2	Inhibits the host serine protease TMPRSS2 to prevent Spike protein‐mediated fusion	Nafamostat is in Phase III clinical trials (NCT04390594).	Nafamostat inhibits the host protease TMPRSS2, thereby blocking SARS‐CoV‐2 membrane fusion and entry in human airway organoids.	[150]
			Camostat mesilate is under investigation in multiple Phase I/II clinical trials for COVID‐19 (NCT04681430, NCT04455815, NCT0435505).	Camostat inhibits SARS‐CoV‐2 infection in lung organoids, thereby validating findings from its clinical trials.	[151]
	ACE2	Recombinant human soluble ACE2 acts as a competitive inhibitor of viral attachment.	hrsACE2 has completed a Phase I/II safety and dose‐finding trial (NCT04335136).	hrsACE2 reduces SARS‐CoV‐2 infection in human vascular and kidney organoids, indicating its therapeutic potential.	[70, 152, 153]
Entry	Endocytosis	Modulates endosomal acidity to inhibit pH‐dependent viral uptake	Niclosamide has entered clinical evaluation (NCT04399356).	Niclosamide inhibits SARS‐CoV‐2 Omicron BA.1 infection in primary human airway organoids, consistent with its mechanism of modulating the endocytic pathway.	[154]
	Spike protein (SARS‐CoV‐2)	Blocks Spike protein‐mediated membrane fusion	Imatinib has been evaluated in a COVID‐19‐related clinical trial (NCT04394416).	Imatinib is a potent inhibitor of SARS‐CoV and MERS‐CoV fusion proteins in hPSC‐Los.	[155, 156]
	NA (neuraminidase)	Inhibits viral neuraminidase, thereby blocking progeny virus release	Oseltamivir, zanamivir, and peramivir are US FDA/EMA‐approved.	Peramivir demonstrates antiviral and neuroprotective efficacy against H3N2 in cerebral organoids, supporting the neural efficacy of NA inhibitors.	[157]
Replication	VP1/VP2 (capsid proteins)	Binds to the hydrophobic pocket of the enterovirus capsid, thereby inhibiting viral uncoating and RNA release	Pleconaril has completed Phase II clinical trials (NCT00031512).	The combination of pleconaril, AG7404, and mindeudesivir protects brain organoids from cell death mediated by EV1, EV11, or CVB5.	[158]
	VP37	Inhibits VP37 to halt virion assembly and transmission.	Tecovirimat is in Phase III clinical trials (NCT05534984).	Tecovirimat inhibits viral production and transcriptional reprogramming in skin organoids, supporting its clinical mechanism.	[131, 159, 160]

*Abbreviations*: ACE2, angiotensin‐converting enzyme 2; COVID‐19, coronavirus disease 2019; CVB5, coxsackievirus B5; EMA, European Medicines Agency; EV1/EV11, echovirus 1/echovirus 11; US FDA, Food and Drug Administration; H3N2, influenza A virus subtype H3N2; hPSC‐LOs, human pluripotent stem cell‐derived lung organoids; hrsACE2, human recombinant soluble angiotensin converting enzyme 2; MERS‐CoV, middle east respiratory syndrome coronavirus; NA, neuraminidase; Nrf2, nuclear factor erythroid 2; SARS‐CoV, severe acute respiratory syndrome coronavirus; SARS‐CoV‐2, severe acute respiratory syndrome coronavirus 2; TMPRSS2, transmembrane serine protease 2; VP1/VP2, viral protein 1/viral protein 2; VP37, viral protein 37.

### 4.2 Applications of Organoids in Oncology

The poor clinical reliability of traditional models has long limited cancer research and precision therapy. PDOs faithfully preserve the heterogeneity, genetic characteristics, and microenvironmental features of primary tumors and have become a transformative tool in oncology. This section focuses on the core applications of organoid technology in oncology, systematically elaborating on its role in exploring carcinogenic mechanisms, personalized drug‐sensitivity testing, predicting metastatic risk, and optimizing clinical trials, with the aim of highlighting its value in overcoming clinical bottlenecks and advancing precision oncology.

#### 4.2.1 Organoid Platforms in Early‐Stage Cancer Research

Early‐stage cancer research has long been constrained by limitations such as the scarcity of patient samples and interspecies differences in animal models, resulting in an incomplete understanding of tumorigenic mechanisms and individualized therapeutic responses. The establishment of organoid models now enables researchers to faithfully recapitulate the dynamic process of tumorigenesis in vitro, providing a reproducible experimental system for investigating early carcinogenic mechanisms.

In gastric organoids, loss of the ARID1A gene induces cellular morphological atypia and activates the FOXM1–BIRC5 signaling pathway, thereby conferring enhanced proliferative capacity and antiapoptotic properties. Notably, these organoids exhibit marked sensitivity to BIRC5 inhibitors [161], suggesting that ARID1A may function as both a driver gene and a potential therapeutic target during early gastric carcinogenesis. Similarly, studies using colorectal cancer organoids have found that dual mutations in the APC and TP53 genes can convert the NOTUM protein from a tumor suppressor to a tumor‐promoting enzyme. Pharmacological inhibition of NOTUM activity can effectively block the progression of adenocarcinoma [162, 163]. Furthermore, the sequential introduction of KRAS and TP53 mutations into pancreatic progenitor organoids has successfully recapitulated the continuous evolution from normal acinar cells to neoplastic structures [164], revealing the dynamic mechanisms of multigene cooperative carcinogenesis. These findings collectively demonstrate the advantage of organoid models in elucidating the mechanisms of early tumorigenesis.

PDOs provide a reliable platform for functional drug‐sensitivity testing. Studies have shown that non‐small cell lung cancer PDOs can predict patient resistance to epidermal growth factor receptor tyrosine kinase inhibitors (EGFR‐TKIs) even before detectable resistance mutations emerge [165, 166], offering critical guidance for preclinical trial design. Additionally, research based on breast cancer PDOs has shown that epithelial–mesenchymal transition (EMT) inhibitors can reverse pre‐existing chemotherapy‐resistant phenotypes [167]. When high‐throughput PDO drug‐sensitivity data are integrated with artificial intelligence algorithms, they enable accurate prediction of drug responses across multiple cancer types [168]. This highlights the potential of organoid technology in advancing functional precision medicine.

Furthermore, breakthroughs in organoid technology within immunotherapy underscore its translational value. In coculture systems of breast cancer PDOs and T cells, researchers discovered that interferon‐induced senescence of CD8^+^T cells is a key mechanism underlying the poor response to PD‐1 inhibitors. Supplementation with nicotinamide adenine dinucleotide precursors can restore T cell cytotoxicity and significantly enhance antitumor immune effects [169], providing a potential metabolic intervention strategy for immunotherapy. Moreover, studies using liver organoid models indicate that a chronic inflammatory microenvironment can promote tumor immune escape by downregulating the expression of major histocompatibility complex class I molecules [170]. This suggests that remodeling of the early tumor‐immune microenvironment (TIME) may be a key initiating event in tumorigenesis, offering novel targets for early intervention to counter immune evasion and improve immunotherapy efficacy.

With their reproducible, manipulable, and clinically relevant characteristics as experimental platform, organoid models are increasingly becoming a core bridge connecting basic research and precision medicine. They provide crucial support for deciphering early tumorigenic mechanisms, validating novel drug targets, and evaluating immunotherapy responses.

#### 4.2.2 Organoid Platforms in Cancer Metastasis Research

Most cancer‐related deaths are closely associated with metastasis. However, traditional model systems struggle to simulate the dynamic and continuous process of tumor metastasis and often fail to adequately recapitulate the human tumor microenvironment. This has led to a significant lag in deciphering metastatic mechanisms and an extremely low success rate in the development of antimetastatic drugs. By leveraging their ability to replicate tumor heterogeneity and model tumor‐microenvironment interactions, PDOs offer irreplaceable value for clinical translation in cancer metastasis investigations.

PDOs provide a high‐fidelity platform for dissecting critical steps in tumor cell invasion and microenvironmental crosstalk. Utilizing microfluidic chip‐based “tumor‐on‐a‐chip” systems, researchers can simulate the complete dynamic process by which tumor cells disseminate from the primary site through the circulatory system to distant organs. Vascularized organoid models constructed on these chips enable continuous monitoring of tumor cell extravasation across the endothelial barrier and subsequent micrometastasis formation. They also offer a convenient platform for evaluating the efficacy of antiangiogenic drugs and EMT inhibitors [171]. Furthermore, immune coculture systems integrating dendritic cells, T cells, and tumor‐associated macrophages with PDOs have been used to elucidate the mechanisms by which tumor‐derived factors suppress immune activation and antigen presentation. Such integrated models provide a powerful platform for studying immune evasion and resistance mechanisms to immunotherapy [172]. PDOs have evolved into a core tool for predicting individualized metastasis risk and screening precise antimetastatic therapeutic agents, directly addressing clinical needs. Studies using colorectal cancer PDOs have shown that LIN28B overexpression activates the PI3K/AKT pathway, significantly enhancing tumor cell invasiveness and liver metastasis potential. In contrast, inhibition of this pathway can reverse these effects [173, 174]. Researchers evaluated the efficacy of a proteolysis‐targeting chimera (PROTAC) targeting PI3K in breast cancer PDOs, confirming its ability to overcome lapatinib resistance. This finding underscores the practical value of PDOs in optimizing targeted therapeutic strategies [175].

#### 4.2.3 Organoid Platforms in Cancer Treatment Research

PDOs effectively simulate individual tumor drug responses, providing a reliable basis for precision medicine and clinical trial enrollment [176]. PDOs are widely utilized for drug screening and for investigating resistance mechanisms in targeted therapy research. A study using breast cancer PDOs compared the efficacy of a PROTAC targeting PI3K with that of traditional inhibitors. The results demonstrated that this PROTAC significantly inhibited the proliferation of resistant cells, reversed lapatinib resistance, and that its efficacy correlated with the patient's PIK3CA mutation status [175]. PDOs analyzed using single‐cell technology and proteomics faithfully recapitulate the tumor ecosystem and accurately predict patient responses to radiotherapy and EGFR inhibitors, showing high consistency with actual clinical outcomes in brain tumor research [177, 178].

Gastric cancer organoids chronically exposed to HER2 inhibitors exhibit compensatory activation of the mesenchymal–epithelial transition proto‐oncogene (MET) signaling pathway in studies of resistance mechanisms and combination therapies. Combining MET inhibitors can restore drug sensitivity, a finding subsequently validated in organoid‐derived xenograft models [179]. Analysis of the drug response index for pancreatic cancer in PDOs revealed that samples with highly activated KRAS signaling pathways are most sensitive to treatment combining poly(ADP‑ribose) polymerase inhibitors (such as olaparib) with gemcitabine. This strategy significantly improved the objective response rate of personalized therapy [180].

Organoids, especially PDOs, have become a core translational tool for advancing basic oncology research and precision clinical treatment. By preserving the inherent genetic heterogeneity, histopathological features, and in situ drug response profiles of primary tumors, PDOs overcome key limitations of traditional preclinical models, such as the lack of tumor microenvironment in 2D cell culture and interspecies differences in xenograft models. Therefore, they support in‐depth analysis of carcinogenic driving mechanisms, identification of specific background resistance mechanisms, and implementation of personalized in vitro drug‐sensitivity testing, and provide a reasonable basis for patient stratification in mechanism‐based clinical trials. Notably, integrating cutting‐edge gene‐editing technology with organoid platforms may further unleash its translational potential. For example, a groundbreaking study recently developed a dual‐switchable yeast‐derived EV platform for the safe and controllable delivery of CRISPR–Cas9 [181]. This technology can achieve precise spatiotemporal control of gene‐editing activity. It is expected to be combined with PDO models to construct or modify specific gene mutations in situ in organoids, thereby directly and dynamically verifying carcinogenic mechanisms and drug resistance in the context of human physiology, and simulating and testing gene editing‐based combination therapy strategies. This pushes research from observation to a new dimension of active intervention and functional verification.

In summary, the continuous improvement of organoid culture systems, especially the deep integration with interdisciplinary technologies such as controllable CRISPR delivery, will systematically address the limitations of these models in functional validation and dynamic intervention, thereby fully realizing their clinical translational potential in precision oncology.

### 4.3 Applications of Organoids in Neurological Diseases

Neurological diseases such as multiple sclerosis (MS), Parkinson's disease (PD), and autism spectrum disorder (ASD) have long faced three core bottlenecks in clinical diagnosis and treatment: unclear mechanisms, inaccurate models, and inefficient drug development. Most candidate drugs for neurological diseases fail in clinical trials due to significant pathological differences between animal models and human conditions. This section introduces the use of organoids in the study of such diseases, highlighting their key role in bridging basic research and clinical applications.

#### 4.3.1 MS

Clinical research on MS faces several critical challenges. Currently, there is a lack of effective treatments for progressive MS (pMS). Existing disease‐modifying therapies are only effective in approximately 30% of patients with relapsing‐remitting MS and cannot reverse neurodegenerative damage. Furthermore, animal models such as experimental autoimmune encephalomyelitis (EAE) mice exhibit significant pathological differences from human MS, leading to the failure of numerous candidate drugs in clinical trials [182, 183, 184].

A team at Roche constructed a coculture system of myelinated human brain organoids and microglia to elucidate the core mechanisms of neuro‐immune interactions and remyelination failure in MS. This model can recapitulate the structure of human brain myelination, with microglia maintaining physiological immune response capabilities and aligning with the microenvironment of the human central nervous system. Experiments confirmed that depletion of microglia significantly reduced the remyelination rate within the organoids. This indicates a crucial role for microglia in myelin repair and provides direct evidence for therapeutic strategies targeting microglial function [185]. Another study introduced peripheral blood mononuclear cells from patients with MS, reflecting peripheral immune status, into lipopolysaccharide (LPS)‐induced neuroinflammatory organoids. This intervention led to a significant increase in the levels of proinflammatory cytokines such as TNF‐α and IL‐6 and a decrease in the fluorescence intensity of the myelin marker myelin basic protein. The fingolimod derivative ST‐1505 effectively reversed the aforementioned inflammatory damage and myelin loss, confirming the utility of this model for screening anti‐inflammatory drugs for MS [186].

A key breakthrough was achieved in research using PDOs derived from patients with the worst prognosis, i.e., primary progressive MS (PPMS). The expression of the cell cycle inhibitor p21 was significantly downregulated in PPMS organoids, leading to impaired oligodendrocyte differentiation. The differentiation rate of oligodendrocytes within the organoids increased from 12% to 27.6% upon exogenous administration of a p21 agonist, suggesting that dysregulation of the p21 pathway may be a core mechanism underlying oligodendrocyte differentiation failure in patients with PPMS [187]. Organoid models are accelerating the screening and translation of candidate drugs. The nano‐oligomer NI12 significantly reduced proinflammatory cytokine levels in neuroinflammatory organoids while upregulating anti‐inflammatory factor expression. Furthermore, NI12 effectively improved motor function and reduced inflammatory cell infiltration in the spinal cord in the EAE mouse model [188]. Based on synergistic validation from organoid and animal models, this drug shows significant translational potential in preclinical studies.

#### 4.3.2 Parkinson's Disease

PD is a neurodegenerative disease characterized by degeneration of dopaminergic neurons in the substantia nigra pars compacta and the abnormal accumulation of α‐synuclein (α‐syn) aggregates [189]. The pathogenesis of PD is complex, involving the interaction of multiple factors such as genetic susceptibility (mutations in genes like LRRK2, PINK1, and SNCA), mitochondrial dysfunction, and neuroinflammation [190, 191, 192]. Traditional animal models cannot accurately replicate the unique human brain structure and pathological progression, presenting significant limitations, especially in exploring early disease mechanisms and drug responses. Midbrain organoids derived from patient‐specific iPSCs can recapitulate key early cellular pathological features of PD, including mitochondrial dysfunction, abnormal energy metabolism, and impaired development of dopaminergic neurons [193, 194]. Researchers observed decreased mitochondrial membrane potential, reduced oxidative phosphorylation efficiency, and weakened synaptic activity in organoid models carrying LRRK2 G2019S, PINK1, or SNCA mutations. These findings suggest that, beyond their known roles in neurodegeneration, such genetic variants may also disrupt cellular energy homeostasis and neural network formation during early neurodevelopment [195, 196, 197]. Further single‐cell transcriptomic and electrophysiological analyses revealed that LRRK2 mutations hinder the morphological maturation and synaptic transmission of dopaminergic neurons, leading to abnormal neural circuit development [196]. These results provide supporting evidence for the “developmental origin” hypothesis of PD, offer a basis for screening early intervention targets, and highlight the unique value of organoids as human‐relevant models for elucidating early disease mechanisms and genetic susceptibility [196]. Midbrain organoids demonstrate significant clinical translational potential for cell‐replacement therapy and can differentiate into mature dopaminergic neurons with electrophysiological activity. These neurons can survive long‐term and integrate into the host neural network after transplantation into PD animal models, thereby improving motor deficits. This outcome confirms the potential of this approach for cell‐replacement therapy [198].

#### 4.3.3 ASD

ASD is a neurodevelopmental disorder characterized by core symptoms including social communication deficits and restricted, repetitive behaviors [199]. ASD is influenced by various genetic and environmental factors, has a complex etiology and exhibits high patient heterogeneity, posing significant challenges for elucidating its pathogenesis and identifying therapeutic targets. Against the backdrop of the continuously rising global prevalence of ASD, there is an urgent need to identify reliable diagnostic biomarkers and effective treatment strategies [200].

Organoid models provide a dedicated tool for precision research in ASD. By differentiating iPSCs derived from patients with ASD, researchers have successfully constructed brain organoid models that can accurately recapitulate key disease‐related phenotypes. For instance, brain organoids carrying SHANK3 gene mutations exhibit abnormal NPC proliferation, delayed neuronal maturation, disrupted microcolumnar structure, and significantly reduced synaptic density compared with healthy controls [201]. Similarly, organoids modeling the 16p11.2 copy number variation deletion show astrocyte overproliferation, which is highly consistent with pathological features observed in patient brain tissue [202]. Single‐cell RNA sequencing validated the reliability of these models, confirming that their gene expression profiles align with transcriptomic differences in specific brain regions of patients with ASD, thereby laying a solid foundation for subsequent mechanistic studies. Brain organoids have revealed abnormalities in key pathways associated with ASD in mechanistic exploration. For example, disruption of the ubiquitination pathway leads to synaptic protein accumulation in models of UBE3A imprinting loss, thereby hindering neural circuit formation [203].

Organoid models have become efficient screening platforms for potential therapeutic drugs for ASD at the clinical translation level. Studies indicate that melatonin can improve neuronal maturation deficits and synaptic dysfunction in SHANK3‐deficient models by modulating the extracellular signal‐regulated kinase pathway [204]. Meanwhile, brain‐derived neurotrophic factor (BDNF) mimetics can inhibit astrocyte overproliferation and promote neuronal survival by activating the TrkB pathway [205]. Furthermore, a recent study [206] found that combining a BDNF mimetic with a mitogen‑activated extracellular signal‑regulated kinase inhibitor synergistically enhances neural network activity in organoids, specifically manifested as a 37.6% increase in the synchrony of calcium ion (Ca^2^^+^) oscillations. This result provides experimental evidence for a combination therapy strategy for patients with 16p11.2 deletion‐associated ASD [207].

We conclude that, as high‐fidelity humanized in vitro models, organoids exhibit unique translational value in virology, oncology, and neuroscience. Although interdisciplinary applications have confirmed the broad potential of organoids, challenges such as the absence of immune microenvironments, incomplete microenvironment simulations, and the lack of unified standards for organoid construction persist. Further technological optimization will expand their clinical applications, and these challenges will be discussed in the following sections.

## 5 Combined Use of Organoids With Other Technologies

**FIGURE 5:**
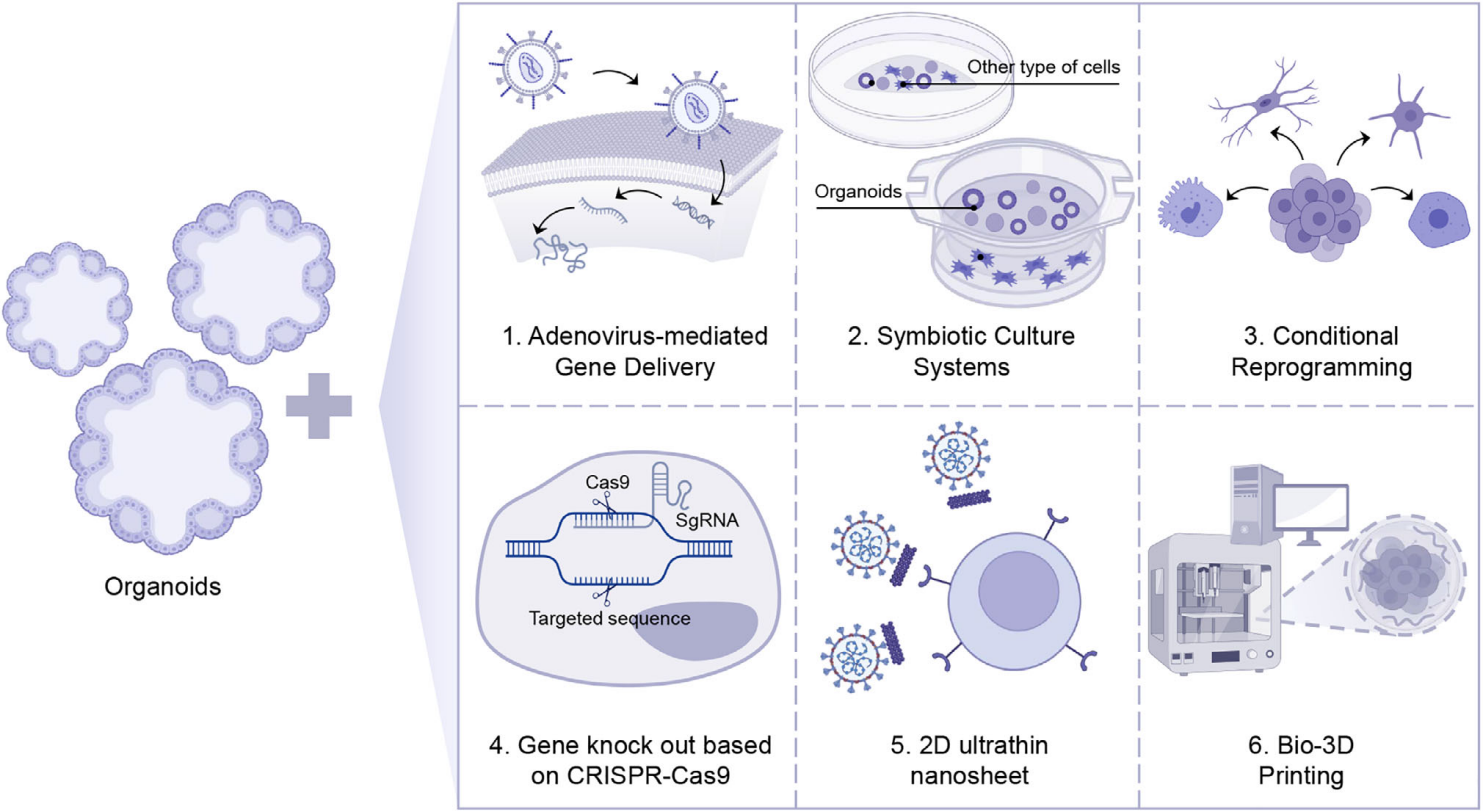
Combination of organoids and other technologies. Organoids can be combined with various technologies, including adenovirus‐mediated gene delivery systems, coculture systems, conditional reprogramming, CRISPR–Cas9 editing, 2D ultra‐thin nanosheets, and 3D bioprinting. The combination of these technologies expands the potential applications of organoids in research and treatment. Created with BioRender.com.

Although organoids play an important role in multiple biomedical fields, independent organoid systems still face inherent limitations, including inadequate assessment of exogenous gene delivery efficiency, incomplete reconstruction of complex tissue microenvironments, and difficulty in in situ gene function analysis. Combining organoids with target technologies has become an important direction for expanding their application value and addressing these limitations. This section focuses on the combined use of organoids and established technologies, including adenovirus‐mediated gene delivery, coculture systems, CRISPR–Cas9, and Bio‐3D printing (Figure 5).

### 5.1 Assessment of Adenovirus‐Mediated Gene Delivery

Adenoviruses, particularly recombinant adeno‐associated virus (rAAV) vectors, have become highly sought‐after tools in gene therapy due to their unique biological properties, offering significant advantages in ocular gene therapy strategies [208].

The low immunogenicity and low cytotoxicity of AAV vectors are key advantages for gene delivery. These properties enable AAV to efficiently deliver therapeutic genes to target cells without eliciting a significant immune response. Additionally, the retina and its accessory tissues, such as the RPE, have relatively low transduction requirements for rAAV, and the eye's immune‐privileged status further enhances the safety and efficiency of gene therapy [209].

AAV vectors offer a high degree of flexibility and customizability through different serotype and promoter combinations. For instance, AAV2‐7m8 demonstrates extremely high transduction rates in retinal organoids, and strong promoters such as CMV and CAG can drive transduction across a wide range of cell types. Moreover, photoreceptor‐specific promoters such as GRK1 can precisely target transgene expression to the outer layer of photoreceptor‐like cells [210]. This precision helps reduce gene expression in nontarget cells and improves treatment effectiveness. Researchers can also continuously modify adenoviral vectors to optimize their performance using organoid models. For example, the transduction efficiency of a novel rAAV based on an engineered AAV capsid with an insertion peptide at position 588 of AAV9 viral protein 1 (VP1) was evaluated using human retinal organoids (HROs), and the results showed that the transduction efficiency of AAV9.NN was superior to that of the parental AAV9 capsid, efficiently transducing retinal neurons and glial cells in HROs. Meanwhile, the NN peptide also enhanced transduction by AAV2 [211].

In summary AAV vectors have become important tools in gene therapy due to their low immunogenicity, low cytotoxicity, high transduction efficiency, and customizability. With ongoing research and optimization using new technologies such as organoids, adenoviral vectors are expected to play a significant role in treating a broader range of diseases, contributing to human health.

### 5.2 Coculture Systems

The human body is a complex organism, with the functions of its organs and systems being interconnected and influencing each other. Single organoid cultures are limited in their ability to simulate the true microenvironment of an organism, which has led to the development of organoid coculture technology. This technique involves culturing organoids alongside other cell types to better mimic the internal human environment in vitro [212].

Coculture systems that combine patient‐derived tumor organoids with immune cells or cancer‐associated fibroblasts have been shown to accurately replicate tumor characteristics, thereby aiding in cancer research and precision medicine more efficiently [213, 214]. Similarly, coculture systems have been constructed to study viral invasion into host cells, providing a more realistic platform for host–virus communication and cell–cell interactions. This approach enables accurate and efficient drug screening, as well as the measurement of targeted antiviral efficacy, mechanisms of drug action, and drug‐induced cytotoxicity and viral replication within host cells in vitro [100].

For instance, researchers have established a reliable in vitro coculture model by coculturing liver organoids with macrophages differentiated from the same hPSCs to investigate the mechanisms of HCV, NAFLD, and host–virus interactions. This multicellular LO model has yielded clinically similar outcomes, with macrophages shifting toward the liver‐resident macrophage type present in vivo, that is, the Kupffer‐like cell type, thereby providing a suitable model for studying the pathogenesis of NAFLD [129]. Additionally, the upregulation of body fat triggered by HCV infection in the host, leading to lipid accumulation in the liver, has been successfully replicated in a LO coculture model, providing a more accurate in vitro model for studying HCV infection mechanisms [129].

Takabe et al. [30] successfully generated iPSC‐derived liver buds (iPSC‐LBs) by mixing iPSC‐derived hepatocytes with human umbilical vein endothelial cells and mesenchymal stem cells in Matrigel. These iPSC‐LBs could connect with host blood vessels after transplantation in vivo, suggesting that the endothelial cells in iPSC‐LBs can be vascularized to stimulate liver organogenesis and regeneration. This model enables high‐throughput drug screening via high‐end imaging readouts of hepatotoxicity and cholestasis [215].

While organoid coculture systems are favored for their ability to reproduce the morphological, physiological, and genetic characteristics of organ tissues in vitro, many challenges remain in establishing reliable coculture models. These include the isolation of additional tissue progenitor cells, the search for nonparenchymal cells, and the establishment of primary organ culture systems that can be used for expansion, including the determination and optimization of amplification and differentiation media [216].

As an emerging technology, organoid coculture needs further practice and exploration to better understand its advantages and shortcomings. This will enable the establishment of various normal and cancerous tissue coculture models with improved stability that can serve patients and society more effectively.

### 5.3 CRISPR–Cas9

The CRISPR–Cas9 system is a groundbreaking gene‐editing tool that has revolutionized the fields of genetic research and therapy. It interacts with DNA via Cas nucleases and, guided by a single‐guide RNA (sgRNA) comprising crRNA and tracrRNA, generates double‐strand breaks in DNA [217]. The CRISPR–Cas9 technology is simple, precise, and efficient, providing an ideal platform for researchers to evaluate gene function without the need for complex in vivo models or ethical constraints.

The CRISPR system can assist in the use of organoids to study the mechanisms of viral infections. In research on coronaviruses, mutant clonal organoids can be generated by transiently transfecting plasmids encoding Cas9–EGFP with sgRNA to evaluate individual host factors related to the replication cycle of SARS‐CoV‐2 [218]. Relevant studies have confirmed in IO mutants that ACE2 and DPP4 are the entry receptors for SARS‐CoV/SARS‐CoV‐2 and MERS‐CoV, respectively, and that the replication of SARS‐CoV‐2 in IOs particularly depends on the cell‐surface protease TMPRSS2 [218]. ADP‐ribosylation factor 6 (ARF6) is a host factor involved in SARS‐CoV‐2 replication at the postentry stage [219]. In addition, the nuclear transcription factor circadian associated repressor of transcription (CIART) has been identified as a key regulator of SARS‐CoV‐2 infection [220]. Deleting CIART can block SARS‐CoV‐2 infection by downregulating the retinoid X receptor pathway. CIART is a transcriptional repressor and a negative regulatory component of the circadian clock, suggesting that the circadian rhythm of host cells may play an important role in viral infections [221, 222]. The CRISPR system can also be used for drug screening. In a study of high‐throughput drug screening using isogenic mutant pancreatic organoids, it was found that perhexiline maleate can maintain the growth of pancreatic cancer organoids carrying kirsten rat sarcoma viral oncogene homolog mutations and primary human pancreatic ductal adenocarcinoma (PDAC) organoids both in vitro and in vivo, and induce apoptosis [223].

The combination of CRISPR–Cas9 technology and organoid models provides a powerful research platform for accurately analyzing complex virus–host interactions during viral infection in physiologically relevant environments. This strategy enables researchers to target and edit key host factors, thereby systematically revealing the molecular mechanisms of key processes such as virus invasion, replication, assembly, and immune escape. However, this system also faces inherent technological challenges. The potential risks of off‐target effects mainly stem from the CRISPR–Cas9 system's inherent tolerance for DNA recognition errors. At the same time, the complex 3D structure and heterogeneous cell composition of organoids may make the distribution and consequences of off‐target events more concealed and difficult to predict than in 2D cells [224]. Heterogeneity in editing efficiency is also an important limitation, as different cell types within organoids differ in cell cycle status, nucleic acid accessibility, and delivery efficiency, making it difficult to achieve uniform gene editing across all target cells and potentially affecting the reliability of phenotypic analysis. This also reflects the necessity of strict validation [225, 226, 227]. It is worth noting that with the continuous development and application of high‐fidelity Cas variants (e.g., SpCas9–HF1, eSpCas9, and HiFi Cas9 [228, 229]), intelligent sgRNA design tools, and systematic off‐target evaluation methods (e.g., CIRCLE‐seq [230, 231, 232, 233]), the accuracy, controllability, and repeatability of CRISPR organoid systems are steadily improving. By further optimizing delivery strategies and editing processes, this platform is expected to play a more critical role in identifying novel host‐dependent factors, evaluating viral pathogenicity, and predicting drug efficacy, thereby accelerating the translation of antiviral strategies from basic research to clinical applications.

### 5.4 Bio‐3D Printing

Bio‐3D printing is a pioneering technology that is transforming traditional biomedical research methods by optimizing 3D cell culture techniques. This innovative approach constructs a 3D digital model of the target organ or tissue using computer‐aided design software or medical imaging data such as CT and MRI scans. The bio‐ink, which comprises cells, biocompatible materials such as hydrogels and collagen, growth factors, and nutrients, is then deposited layer by layer onto the construction platform according to the digital model, controlled by a sophisticated mechanical system [234]. Seasonal influenza A virus (IAV) infection has been successfully modeled using 3D‐printed cellular hydrogels. In these bioprinted lung tissue models, a nonuniform aggregated infection pattern, efficient viral replication (H3N2, Pan/99), and an anti‐inflammatory response by IL‐29‐secreting epithelial cells were observed, closely resembling those found in IAV‐infected human lungs [235, 236]. Bioprinted 3D models are also facilitating clinical studies and drug screening. For instance, 3D bioprinting that replicates nonuniform tumor regions in vivo using tumors derived from patients with primary colorectal cancer has demonstrated that long‐established microtumors retain proliferating cells, as well as areas of necrosis and hypoxia. Hypoxic zones are crucial for the success of chemotherapy because hypoxia mediates several mechanisms associated with therapeutic resistance. The validity of this model as a useful screening tool was demonstrated using a late‐stage oncolytic virus derived from thymidine kinase/ribonucleotide reductase‐deficient cowpox virus [237].

Furthermore, bio‐3D printing technology has great potential for studying viral infection mechanisms. A natural 3D oBRB tissue (3D‐oBRB) was designed by bioprinting endothelial cells, pericytes, and fibroblasts on the basal side of a biodegradable scaffold and building an RPE monolayer on top. Researchers used an anti‐VEGF monoclonal antibody (bevacizumab) to treat wet age‐related macular degeneration and performed experiments on 2D‐iRPE and 3D‐oBRB, validating 3D‐oBRB as a physiologically relevant platform applicable for clinical and drug discovery [238]. Additionally, 3D bioprinting can be used to study viral infection mechanisms. In a study on the pathogenesis of IAV, human small airway epithelial cells were cultured on chitosan/collagen scaffolds and infected with IAV subtypes H1N1 and H3N2 at the air–liquid interface. Primary epithelial cells in the 3D model expressed significant amounts of aquaporin‐5 and cytokeratin‐14 compared with those in a 2D model of lung tissue [239]. In another study, human HepaRG (hepatic cell line) hepatocytes were printed using a bio‐ink comprising alginate, gelatin, and human extracellular matrix (hECM) for transduction of AAV vectors and for the study of human adenovirus 5 infection. AAV vectors of serotype 6 efficiently transduced a tissue model in which the shRNA encoded by the AAV expression cassette successfully silenced the endogenous target (procyclin B) [240].

The development of bio‐3D printing technology still faces multiple challenges. First, there is an inherent contradiction between printing accuracy and efficiency, as tissue function highly depends on its fine microstructure. The resolution of mainstream extrusion bioprinting is usually 50–300  µm, making it difficult to directly construct capillary networks [241]. Although photopolymerization‐based methods can improve the resolution to 10–50 µm [234, 242, 243], the printing speed often decreases significantly to reduce the impact of shear force on cell activity, which limits the practical feasibility of large‐scale tissue manufacturing [244]. Second, the material science bottleneck of bio‐ink is prominent. The ideal bio‐ink needs to balance good printability, excellent cell compatibility, and long‐term structural stability. However, there is currently a lack of universal materials that can fully meet these requirements, which constitutes a continuous core challenge in this field. In addition, existing research generally focuses on structural imitation and neglects functional verification. A large amount of work focuses on the simulation and construction of morphology, but it lacks systematic evaluation of long‐term tissue functional maturity in vitro, in vivo immune compatibility, host integration dynamics, and physiological response authenticity [245]. The lack of functional validation significantly increases the uncertainty of the pathway for translating research results into clinical practice.

In summary, bio‐3D printing technology offers broad application prospects in biomedical research, with unique advantages. As technology continues to advance, bio‐3D printing will bring greater benefits to human health.

### 5.5 Conditional Reprogramming

Organoid technology can also be combined with conditional reprogramming (CR) technology, and significant progress has been made in kidney research in recent years. Using CR and organoid technology, a long‐term culture system for normal human kidney proximal tubule epithelial cells (has been successfully established [246]. CR helps maintain the differentiation potential of cell lines, DNA repair capacity, and the expression of cell‐type‐specific functional markers, including ACE2 protein [247]. These cultured cells provide a new platform for studying physiological cell models and can be used to examine the relationship between SARS‐CoV‐2 and renal function, the innate immune response of renal cells to the virus, and for drug development and safety evaluation.

Collectively, these integrated strategies have synergistically enhanced the biological fidelity, analytical depth, and translational reproducibility of organoid models, expanding their application scope from basic mechanistic research to preclinical validation and the development of personalized medicine. The technical frameworks outlined in this chapter provide a foundational basis for the iterative optimization of organoid systems and lay the groundwork for addressing complex context‐dependent biological questions and accelerating the clinical translation of organoid‐based research.

## 6 Limitations of Organoids and Bottlenecks in Clinical Translation

Despite the application of organoids to advance research in fields such as virology, oncology, and neurological diseases, translating organoid technology into clinical applications remains challenging compared with its rapid development in basic research. The following analysis will address the obstacles to the clinical translation of organoids.

### 6.1 Defects in Simulating the 3D Microenvironment and Multicellular Interactions

#### 6.1.1 Systemic Deficiencies in Reconstructing the Immune Microenvironment

The immune microenvironment plays a pivotal role in the onset, progression, and treatment response of various diseases. However, current organoid models still have limitations in systematically reconstructing their complexity. In virology research, the complex interactions between viruses and host immune cells are key to understanding viral pathogenesis and developing antiviral strategies. SARS‐CoV‐2 variants can evade immune clearance by escaping neutralizing antibodies, making it particularly crucial to simulate the recruitment, activation, and functional dynamics of immune cells during viral infection [248].

The TIME is a highly complex and dynamic system composed of cancer cells, stromal cells, immune cells, the ECM, and signaling molecules. Among these, the intricate interactions between immune cells have a decisive impact on tumor progression and therapeutic response [249, 250]. Traditional 2D cell cultures and animal models struggle to capture this complex spatial heterogeneity and dynamic nature accurately [251]. For instance, the unique immune dysfunction in glioblastoma (GBM) leads to its resistance to immunotherapy, which is closely linked to the complex interactions within the tumor microenvironment [252]. The classification of TIME into immune‐inflamed, immune‐excluded, and immune‐desert types in colorectal cancer research reveals differences in key features such as immune cell infiltration levels, neoantigen expression, and PD‐L1 expression, thereby influencing the response to immunotherapies [249, 253].

In neurodegenerative diseases, alterations in the brain's immune microenvironment and cellular dynamics play a key role in disease progression [254]. For example, understanding the impact of myeloid cells on the brain's immune microenvironment in sepsis‐associated encephalopathy, as well as changes in glial cells, requires organoid models that can simulate these complex cellular communication networks. However, accurately recapitulating the permeability of the blood–brain barrier (BBB) and the dynamic interactions between immune cells and neurons in vitro remains a significant challenge [252].

#### 6.1.2 Lack of Functional Vascularization and Associated Barriers

Tumor growth and metastasis depend heavily on angiogenesis [255]. Hypoxia, a prevalent feature of the tumor microenvironment, promotes tumor progression and therapy resistance and regulates tumor cells and the surrounding microenvironment through EV‐mediated intercellular communication [256]. Radiotherapy is a primary cancer treatment, but tumor cell radioresistance limits its clinical efficacy. Hypoxia and metabolic reprogramming are hallmarks of tumorigenesis and progression, closely associated with radioresistance [257]. Therefore, organoid models lacking functional vascularization cannot accurately simulate hypoxic gradients and nutrient delivery within tumors, leading to inaccurate assessments of treatment response [256]. For instance, drug resistance in PDAC is partly attributed to insufficient vascularization and the formation of a fibrotic stroma in its microenvironment [258]. Additionally, tumor‐associated neutrophils can sustain tumor angiogenesis and promote tumor cell proliferation and spread [249].

Viral spread within the body often occurs via the circulatory system. In vitro models lacking functional vasculature struggle to simulate viral dissemination in the bloodstream, virus–endothelial cell interactions, and vascular inflammatory responses triggered by infection [248]. The BBB is crucial for maintaining brain homeostasis [255]. In neurodegenerative diseases and brain tumors, BBB integrity is often compromised [252, 259]. The absence of functional vascularization means organoid models cannot simulate the structure and function of the BBB, thereby hindering the effective study of important pathophysiological processes such as drug delivery, immune cell infiltration, and ischemia–reperfusion injury [252]. For example, in GBM therapy research, models lacking vasculature make it difficult to evaluate the delivery efficiency of nanomedicines across the BBB.

New strategies are needed to form stable, functionally normal vascular networks within organoids [255, 259]. These include coculture systems, organ‐on‐a‐chip technologies, and matrix engineering techniques. By coculturing organoids with immune cells or fabricating organ‐on‐a‐chip devices, aspects of the immune system can be introduced while enabling dynamic process monitoring during experiments. This enables the recreation of in vivo metabolic or immune interactions, yielding test data that are closer to the human context. Furthermore, matrix engineering techniques can adjust the composition of the ECM used in organoid culture to simulate the TIME, thereby recreating the tumorigenesis process in vitro.

### 6.2 Standardization and Reproducibility

The process of generating organoids involves various starting cell sources, different medium formulations, and choices of extracellular matrices (e.g., Matrigel), all of which can lead to variability in organoid morphology, function, and molecular profiles [260]. Even when laboratories use similar protocols, minor operational variations can yield organoids with significantly different functional properties. This lack of standardized generation protocols makes it difficult to effectively compare and integrate organoid data across laboratories and research projects [260].

Organoid culture is a dynamic process with strict requirements for medium composition, oxygen concentration, pH, mechanical stimuli, and scaffold physicochemical properties. Currently, there is no unified culture system applicable to all types of organoids, leading each research team to optimize culture conditions based on their own needs [260]. While this diversity has, to some extent, fostered the development of specific organoid models, it also increases the difficulty of overall research reproducibility. Even for the same type of organoid, different culture environments can lead to variations in differentiation trajectories, cellular composition, and maturity, thereby affecting the accuracy and reliability of experimental results [260]. Simultaneously, organoid research has yet to establish a universal, widely accepted set of quality control standards for assessing organoid maturity, functionality, and similarity to in vivo tissues [260]. Existing quality assessment methods often rely on histomorphological observation, immunofluorescence staining, and gene expression analysis. These methods can be subjective and vary between laboratories, and data analysis and interpretation also require standardization [252].

Industry and academia should collaborate to develop a unified and detailed plan for organoid generation and cultivation and encourage researchers to strictly adhere to these standards to address these issues. Meanwhile, establishing unified quality control standards and biomarkers is crucial for ensuring the quality and consistency of organoid models. In addition, the introduction of automated equipment and high‐throughput screening technology can reduce human operational errors and improve the standardization and repeatability of organoid preparation and analysis. Notably, the preparation of engineered stem cells through synthetic biology and gene editing can introduce controllable genetic modifications at the source to ensure consistent differentiation and reliable responses. At the same time, artificial synthetic hydrogels with defined compositions and adjustable physical properties can be used to replace natural matrices, which can build a well‐defined extracellular microenvironment for organ‐like cells and enable the construction of a highly controllable, repeatable, traceable “organoid factory,” laying a solid engineering foundation for meeting regulatory requirements and achieving high‐throughput clinical applications [261].

### 6.3 Regulatory Pathway and Cost‐Effectiveness

The clinical translation of organoid technology remains constrained by the dual challenges of regulatory alignment and cost‐effectiveness. From a regulatory perspective, while organoids have gained recognition as part of new approach methodologies by agencies including the US FDA and EMA, and their data are increasingly accepted in nonclinical toxicity studies and mechanistic investigations, these models have not yet been systematically integrated into the core decision‐making framework for drug evaluation [262, 263]. In practice, evidence derived from organoids is often used to elucidate mechanisms of action or to complement traditional animal studies in support of investigational new drug applications; however, it seldom serves as a pivotal basis for regulatory approval. This gap highlights the absence of a harmonized validation framework and consistent technical standards for human‐relevant organoid models within regulatory science. Moving forward, establishing reproducible performance benchmarks and quality control guidelines will be essential to advance organoids from a supplementary tool to a decisive component in regulatory submissions.

Regarding cost and scalability, organoid culture remains resource‐intensive, relying on expensive matrices, tailored cytokine cocktails, and prolonged differentiation protocols. Consequently, per‐sample preparation and analytical costs are substantially higher than those associated with conventional 2D cell cultures. Although organoids, particularly PDOs, hold significant promise for predicting patient‐specific drug responses and reducing the attrition rate of preclinical animal studies [47], current methodologies cannot readily support large‐scale, high‐throughput screening demands. Therefore, broader clinical adoption will depend on further validation of their biological predictive power and engineered innovations that enhance feasibility. These include automated culture platforms, microfluidic integration, the development of defined, scalable culture matrices, and standardized phenotypic readouts. Only through such integrated advances can a clear, scalable translation pathway be established that satisfies both regulatory expectations and economic sustainability.

## 7 Conclusion and Prospects

This review systematically examines the key applications of organoid technology in virology, oncology, and neurological disease research, highlighting its value as a novel in vitro model that bridges basic research and clinical translation. Organoids provide a crucial platform for in‐depth investigation into viral replication mechanisms and tissue tropism. This technology has been successfully applied to study the replication kinetics of SARS‐CoV‐2 and enteroviruses, establish stable in vitro replication models for NoV, elucidate ZIKV neuropathogenesis and placental infection, and provide new insights into the complete life cycle of HBV and the replication process of HRV‐C. Furthermore, organoids serve as efficient research tools for vaccine development and antiviral drug screening for viruses such as RSV and HSV. In oncology research, PDOs can highly recapitulate tumor heterogeneity and drug response features. This model provides a high‐fidelity platform for deciphering early carcinogenic mechanisms and identifying drug‐resistance‐related targets (e.g., ARID1A, NOTUM, and PI3K signaling pathways) and offers advantages in personalized drug‐sensitivity testing, metastasis risk prediction, and preclinical studies, advancing the practice of precision oncology. For neurological diseases, models such as brain and midbrain organoids have reproduced core pathological features, including demyelination in MS, dopaminergic neuron damage in PD, and neurodevelopmental abnormalities in ASD. They provide specialized tools for exploring early disease mechanisms, studying neuro‐immune interactions, and conducting drug screening.

The core advantages of organoid technology lie in its patient specificity, high degree of tissue mimicry, and experimental manipulability. It effectively compensates for the limitations of traditional 2D cell cultures, which lack a microenvironmental support, and of animal models, which exhibit interspecies differences, thereby enhancing the potential for translating research findings to the clinic. For instance, the recapitulation of early pathological features by neural organoids has opened new avenues for identifying therapeutic targets for refractory diseases such as MS and ASD. However, the technology still faces challenges such as insufficient standardization, limited availability of immune cells and vascular structures, long culture periods, and high costs, which limit its widespread application in clinical and large‐scale research settings.

In the future, the development of organoid technology should focus on three major directions: precision, integration, and clinical translation. On the technical front, standardization and automation of culture systems are needed to reduce operational costs and improve experimental reproducibility. Regarding functional expansion, integration of multidisciplinary technologies such as coculture systems, microfluidic chips, single‐cell sequencing, and artificial intelligence is essential to develop integrated platforms such as organoid‐on‐a‐chip, multicomponent interaction models, and AI‐assisted prediction systems. This will enhance physiological relevance and research efficiency. In terms of clinical translation, efforts should be accelerated to incorporate organoid technology into clinical pathways, for example, by establishing clinical guidelines for PDOs based on drug‐sensitivity testing to promote its routine use in personalized cancer therapy and rare disease diagnosis.

In summary, organoid technology has become a vital tool in biomedical research, with broad applications in disease mechanism elucidation, drug development, and precision medicine. Although technical bottlenecks remain, continued in‐depth research and technological optimization will enable organoids to play an increasingly important role in addressing major health challenges such as viral infections, malignant tumors, and neurodegenerative diseases, advancing modern medicine toward a more individualized and precise future.

## Author Contributions

HF, BH, and XL designed the research. KL, RC, ML, and ZT reviewed the literature. HF, BH, XL, KL, RC, and ZT wrote and revised the manuscript. All authors contributed to the article and approved the submitted version.

## Conflicts of Interest

The authors declare no conflicts of interest.

## Ethics Statement

The authors have nothing to report.

## Data Availability

The authors have nothing to report.
